# Emotion regulation strategy use and daily levels of affective arousal, affective valence and dissociation in a clinical sample

**DOI:** 10.1038/s41598-026-70906-7

**Published:** 2026-09-15

**Authors:** Martin Tegtmeyer, James J. Gross, David A. Preece, Johannes B. Heekerens

**Affiliations:** 1 Department of Psychiatry and Neurosciences, Charité – Universitätsmedizin Berlin, Berlin, Germany; 2 Department of Education and Psychology, Free University Berlin, Berlin, Germany; 3 Department of Psychology, Stanford University, Stanford, USA; 4 School of Population Health, Curtin University, Perth, Australia; 5 School of Psychological Science, The University of Western Australia, Perth, Australia; 6 Medical Faculty Mannheim, Department of Psychosomatic Medicine and Psychotherapy, Central Institute of Mental Health, Heidelberg University, Mannheim, Germany

**Keywords:** Emotion regulation, Dissociation, Affect, Ecological momentary assessment, Borderline personality disorder, Posttraumatic stress disorder, Neuroscience, Psychology, Psychology

## Abstract

Clinical models and cross-sectional research suggest that habitual use of disengagement-oriented emotion regulation strategies is associated with high levels of affective arousal, negative valence, and dissociation, whereas engagement-oriented strategies show the opposite effect. However, intensive longitudinal evidence remains scarce. This study examined whether habitual use of 10 emotion regulation strategies, assessed using the Process Model of Emotion Regulation Questionnaire (PMERQ), predicts affective arousal and valence, and dissociation in daily life. We analysed ecological momentary assessment data from 88 individuals with posttraumatic stress disorder, borderline personality disorder, and dissociative disorders. Baseline PMERQ scores did not robustly predict daily levels of affective arousal and valence, and dissociation. The overall null findings of our study could reflect either a true absence of meaningful effects, insufficient sample size, or a mismatch between trait-level emotion regulation measures and momentary affect/dissociation. Future studies should prioritise momentary assessments of emotion regulation to disentangle state and trait effects.

**Supplementary Information:**

The online version contains supplementary material available at https://doi.org/10.1038/s41598-026-70906-7.

## Introduction

Difficulties with emotion regulation characterise many mental disorders^1^. Emotion regulation refers to both intentional and automatic strategies that influence the intensity, duration, and expression of emotion^2,3^ The use of different emotion regulation strategies has been shown to contribute to or protect from psychopathology in various ways (see^1^ for a review). Two transdiagnostic manifestations of psychopathology are elevated levels of negative affect and dissociation^1,4,5^. Affect is often mapped across the two dimensions of arousal (i.e., feeling tense vs. calm) and valence (i.e., feeling good vs. bad;^6^. Dissociation is broadly characterised as a disruption in the integration of consciousness, perception, memory, emotion, identity, motor control, body representation, and behaviour ^7,8^. Two common forms of dissociation are derealisation, which involves altered perceptions of the external world, and gaps in awareness, limiting the ability to access information and mental control functions^7,8^. Previous studies from daily life found that increased affective arousal and negative valence co-occurred with high levels of dissociation in the same moment^9–11^. However, to our knowledge, no study has yet investigated the role of emotion regulation difficulties predicting daily dynamics of affective arousal and valence, and dissociation. This study addresses this gap. We begin by introducing a widely used emotion regulation framework, followed by brief overviews of affect and dissociation, and then review studies linking emotion regulation to these phenomena.

A widely adopted framework for systematically understanding emotion regulation is the Process Model of Emotion Regulation (see Figure S1 in the Supplementary Material for an illustration)^3,12^. The process model posits that emotion regulation can occur along five stages of the emotion-generative process: situation selection, situation modification, attentional deployment, cognitive change, and response modulation. Within each stage, a distinction can be made between engagement-oriented and disengagement-oriented strategies. While the adaptiveness of a strategy can be context-dependent^13^, engagement-oriented strategies are generally seen as adaptive and aim to approach key features of a specific stage (e.g., asking a colleague directly after a meeting: “Can we talk for five minutes? I felt like we were not on the same page.”), whereas disengagement-oriented strategies are seen as generally maladaptive and focus on avoiding them (e.g., saying “everything’s fine” when one’s partner asks if something is wrong, even though one is upset)^14,15^. Cross-sectional evidence supports that the use of engagement-oriented strategies is associated with indicators of mental health (psychological well-being, life satisfaction, positive affective valence) and disengagement-oriented strategies are associated with indicators of mental illness (anxiety, depression, negative affective valence)^15,16^.

Negative affect is a meaningful indicator of psychopathology^1,17^. From a functional perspective, emotions are adaptive when appropriately matched to context, duration, and intensity, but when affective arousal combined with negative valence become excessively intense, prolonged, or frequent, they can contribute to significant distress and impairment^1^. High levels of affective arousal together with negative valence are closely tied to maladaptive patterns including rumination, cognitive distortions, and interpersonal dysfunction, which maintain and self-reinforce symptoms^18^. Contemporary research highlights the need for time-intensive, context-sensitive methods to capture daily affective dynamics and their role in psychopathology^18^. For instance, ecological momentary assessment studies showed that high levels of affective arousal in combination with negative valence are associated with strong dissociation^9–11^.

Dissociation is a clinically relevant phenomenon, frequently observed in patient populations and linked to clinically urgent outcomes, such as self-harm and suicidality^19–21^. It is prevalent across a range of mental disorders, including the dissociative subtype of posttraumatic stress disorder (PTSD), borderline personality disorder (BPD), and dissociative disorders (DD)^21,22^. While trauma is an established risk factor for dissociation, many individuals with dissociative symptoms report neither trauma nor neglect, and far less is known about other distal and proximal antecedents of dissociative states^20,21,23–25^. Current research highlights emotion regulation difficulties as a potential contributing factor to dissociation^26,27^.

### Linking emotion regulation to affective arousal, affective valence, and dissociation

Emotion regulation difficulties are associated with heightened affective arousal and negative valence in daily life, as demonstrated in a recent meta-analysis^28^. Specifically, the authors linked the use of maladaptive strategies, such as rumination and suppression, to high levels of affective arousal and negative valence both concurrently and prospectively. Adaptive strategies, including reappraisal and acceptance, were associated with high levels of positive valence in daily life. Although most studies included in the meta-analysis focused on community samples, evidence from clinical groups supports similar patterns: individuals with PTSD and BPD both show links between emotion regulation deficits and more affective arousal and negative valence in daily life^29,30^.

Beyond affective arousal and negative valence, emotion regulation difficulties also appear to predispose individuals to dissociation, supported by two recent meta-analyses based on cross-sectional work^26,27^. Overall, both studies report a medium-sized association between emotion regulation difficulties and dissociation. The use of maladaptive emotion regulation strategies was linked to the highest levels of dissociation, with effect sizes remaining equivalent across clinical and non-clinical samples. These findings are broadly in line with accounts of dissociation as a largely automatic and involuntary avoidance response that may allow temporal relief from emotionally overwhelming experiences^31^. However, existing studies are predominantly cross-sectional and focus on broad orientations of emotion regulation (for cross-sectional reviews see^20,26,27^, for exceptions see^32,33^.

Importantly, affective arousal and negative valence, and dissociation are not only linked to emotion regulation difficulties but also appear to influence each other, as cross-sectional research has linked elevated affective arousal and negative valence with increased levels of dissociation^20,34,35^. Additionally, evidence from daily life demonstrated that increases in affective arousal and negative valence temporally precede depersonalisation and derealisation^10,32^. These temporal dynamics could be influenced by the use of specific emotion regulation strategies^27^. One longitudinal data study demonstrated that the use of engagement-oriented affect regulation strategies predicted low levels of dissociation two months later, whereas the use of disengagement-oriented strategies was linked to high levels of dissociation at that same 2-month time-point^33^. Another recent ecological momentary assessment study found that the use of engagement- and disengagement-oriented emotion regulation strategies did not predict subsequent daily levels of affective valence or negative valence or dissociation in a clinical sample^32^. However, this study did not disentangle the effects of individual strategies, which is an important limitation that we aim to address in the present study.

### The present study

The objective of the present study was to investigate the links between the habitual use of 10 individual engagement- and disengagement-oriented emotion regulation strategies and affective arousal and valence and dissociation in daily life in a clinical sample. Building on the Process Model of Emotion Regulation, we investigated emotion regulation strategies across five stages of the emotion-generative process, extending beyond mere cognitive appraisal. We employed a well-established research design in which these trait-level emotion regulation tendencies are used to predict momentary outcomes in daily life. We expected that individuals who relied heavily on disengagement-oriented emotion regulation strategies would show higher levels of affective arousal and more negative affective valence than individuals who used more engagement-oriented and fewer disengagement-oriented strategies. Particularly, the use of disengagement-oriented strategies during the stages of attentional deployment, cognitive change, and response modulation were anticipated to predict high levels of affective arousal and negative valence.

We further expected the use of disengagement-oriented strategies to predict high levels of dissociation across its derealisation and gaps in awareness facets (i.e., we do not expect differential effects for these two facets of dissociation). Specifically, we expected disengagement-oriented strategies used at the stages of situation modification, attentional deployment, and cognitive change to relate to high levels of derealisation and gaps in awareness in daily life, supporting the role of dissociation as a mechanism of automatic avoidance. Finally, we expected that the use of disengagement-oriented strategies would strengthen the temporal persistence of dissociation and affective arousal and negative valence across time, while engagement-oriented strategies would buffer against such persistence.

## Methods

This study utilises data from a previously published study^32^. The sample, baseline measures and Ecological Momentary Assessment data of affective arousal and valence, and dissociation were collected for the original study. That study examined the temporal interplay between affective arousal and valence, and dissociation in terms of within-person means, contemporaneous, autoregressive, and cross-lagged effects. Beyond that, the study reported associations for the broader emotion regulation dimensions of engagement- and disengagement-oriented dimensions predicting these within-person means and temporal effects. The novel contribution of the present study is the fine-grained examination of the ten individual emotion regulation strategies from the Process Model of Emotion Regulation as between-person predictors for the previously reported parameters of affective arousal and valence, and dissociation. The current study was not pre-registered. All analytical decisions are documented under https://doi.org/10.17605/OSF.IO/8X3QP to ensure transparency.

### Participants

In this study, we reanalysed data from a previous investigation that focused on the temporal interplay of affective arousal and valence, and dissociation to better understand the way the habitual use of individual emotion regulation strategies influences these processes^32^. Our sample included adults meeting the DSM-5 criteria for PTSD, BPD, and/or DD, as these groups show the highest levels of dissociation^5,36^. Structured interviews were used to confirm the diagnoses and rule out any current major depressive disorder, and lifetime bipolar or psychotic disorder^37–39^. Participants had to report a sum score of at least 20 on the German translation of the Dissociative Symptoms Scale (DSS^40^ or of at least 5 on the Depersonalisation/Derealisation or Gaps in Awareness subscales of the Brief Dissociative Symptoms Scale (DSS-B^41^ to ensure frequent experience of dissociative symptoms during the study period. Exclusion criteria included the diagnoses of bipolar disorder, psychotic disorders, severe depression (≥ 8 symptoms), anorexia nervosa, recent severe alcohol or substance use disorders. Participants on stable psychotropic medication for at least 2 weeks were included while medication type and dosage were documented. Participants were not included if they were currently receiving inpatient treatment. A non-clinical group was not included due to potentially insufficient dissociation^10,27^.

An overview for all the demographics and diagnoses is provided in Tables S1 and S2 in the Supplementary Material. Among the 229 patients assessed for eligibility, 88 participants met the inclusion criteria and formed our sample. Forty-six participants were diagnosed with PTSD, 63 of them were diagnosed with BPD, and four of them were diagnosed with Depersonalisation/Derealisation disorder. Participants were between 19 and 53 years old, with a mean age of 30.3 years. Sixty-seven people identified as female individuals, 10 people identified as male individuals, 9 people identified as non-binary, 1 person identified as other, and 1 person did not specify. This distribution aligns with the demographic composition of populations on clinical wards^19^. Sixty-one participants completed school with university entrance qualification or advanced technical college entrance qualification. Seventy-three patients identified as white or Caucasian. Forty-seven participants were receiving outpatient psychotherapy, 44 participants were medicated with antidepressants, 19 participants were medicated with antipsychotics, 8 participants were medicated with anxiolytics, 5 participants were medicated with stimulants, and 6 participants were medicated with other psychotropic drugs.

### Power analysis

The sample size for the original research project was computed a priori using Monte Carlo Simulations^42^ in M*plus*^43^, with models generally comparable to our analyses. Concerning the specific research question of this study, we retrospectively assessed the power with additional simulations using estimates for model parameters from our data (such as factor loadings, intercepts, and variances). Bias was considered acceptable if the simulated point parameter estimate of the effect of interest was within 5% of the population value. Power was set at least 80%, and the analyses had to avoid errors, such as convergence failures or improper solutions^44^.

For our analysis, we considered the smallest uncorrected yet significant effect from our main analysis that was small-to-medium in size (standardised estimate = − 0.25, which is equivalent to 6.3% explained variance). We specified an alpha of 0.05, *N* = 87, *T* = 84, with a compliance rate above 90%, and the remaining parameter estimates obtained from our data. These criteria resulted in a power of 0.60 and a relative difference of 3.4% between simulation (unstandardised estimate = 0.16) and the population parameter estimate (unstandardised estimate = 0.15). This result illustrates that our models’ bias within the parameter estimation can be deemed acceptable, while statistical sensitivity was potentially limited for small and small-to-moderate between-person effects (statistical code and details available at https://doi.org/10.17605/OSF.IO/8X3QP).

### Procedures

Participants were recruited via the Department of Psychiatry and Neurosciences at Charité – Universitätsmedizin Berlin and through social media. Participants were first invited for baseline diagnostics, followed by the assessment in daily life (see Table S3 in the Supplementary Material for a summary of the data collection process). All patients gave written informed consent, and participation was entirely voluntary. The project was approved by the Charité ethics committee following the Declaration of Helsinki (EA4_062_22). All methods were performed in accordance with relevant guidelines and regulations.

### Baseline measures

To measure the habitual use of 10 emotion regulation strategies, we employed the German version of the Process Model of Emotion Regulation Questionnaire (PMERQ^16^. Participants rated 45 statements on a scale ranging from 0 (*strongly disagree*) to 6 (*strongly agree*). Descriptive statistics (Table 1) and predictive effects for the broad engagement and disengagement dimensions (second-order factors) have already been reported in an earlier study^32^. As a unique contribution to this study, we examined results for the 10 individual PMERQ emotion regulation strategies separately (first-order factors). The PMERQ strategies reflect the five stages of the emotion-generating process as well as engagement- or disengagement orientations (also see Figure S1 in the Supplementary Material). These 10 strategies include *Confront Unpleasant Situations*, *Avoid Unpleasant Situations*, *Resolve Conflicts*, *Avoid Conflicts*, *Focus Elsewhere*, *Cognitively Distract*, *Consider Benefits*, *Reduce Importance*, *Emotional Sharing*, and *Expressive Suppression*.

**Table 1:** Latent intraclass-correlations, means, standard deviations, and reliability estimates for the habitual use of emotion regulation strategies at baseline, as well as affective arousal, affective valence, and dissociation in daily life.

	ICC	95% CI	Mean	95% CI	SD	95% CI	Reliability	95% CI
*Habitual Use of Emotion Regulation Strategies at Baseline (PMERQ; N = 87)*								
Confront Unpleasant Situations	-	-	3.31	[2.97, 3.65]	0.92	[0.51, 1.20]	0.86	[0.78, 0.90]
Avoid Unpleasant Situations	-	-	4.31	[3.74, 4.89]	1.19	[0.29, 1.65]	0.88	[0.81, 0.92]
Resolve Conflicts	-	-	4.24	[3.92, 4.55]	0.71	[0.00, 1.05]	0.88	[0.82, 0.92]
Avoid Conflict	-	-	3.63	[2.97, 4.00]	1.14	[0.80, 1.40]	0.89	[0.83, 0.93]
Focus Elsewhere	-	-	2.86	[2.50, 3.21]	0.65	[0.00, 0.95]	0.74	[0.60, 0.83]
Cognitively Distract	-	-	4.27	[3.97, 4.58]	0.73	[0.00, 1.03]	0.84	[0.77, 0.90]
Consider Benefits	-	-	2.97	[2.66, 3.27]	0.97	[0.64, 1.20]	0.89	[0.83, 0.93]
Reduce Importance	-	-	3.26	[2.83, 3.68]	1.39	[0.91, 1.75]	0.85	[0.60, 0.92]
Emotional Sharing	-	-	3.45	[3.13, 3.77]	0.98	[0.57, 1.25]	0.82	[0.73, 0.88]
Expressive Suppression	-	-	4.27	[3.67, 4.88]	0.34	[0.00, 1.17]	0.68	[0.03, 0.80]
*Affect in Daily Life (MDMQ; N = 88)*								
Arousal	0.27	[0.18, 0.39]	3.06	[2.89, 3.23]	0.71	[0.58, 0.84]	0.83	[0.82, 0.83]
Valence	0.39	[0.28, 0.53]	2.87	[2.68, 3.07]	0.87	[0.71, 1.00]	0.82	[0.82, 0.83]
*Dissociation in Daily Life (DSS-B; N = 88)*								
Derealisation	0.41	[0.30, 0.54]	1.15	[1.02, 1.29]	0.59	[0.51, 0.72]	0.83	[0.82, 0.84]
Gaps in Awareness	0.42	[0.27, 0.56]	1.07	[0.94, 1.21]	0.58	[0.51, 0.73]	0.80	[0.79, 0.81]

Note. Reliability coefficients for PMERQ subscales, as well as data for affect and dissociation was previously reported previously^32^; other data are novel contributions. Intraclass correlations (ICCs), means, standard deviations (SDs), and bivariate correlations are based on Bayesian estimates. Affective arousal and valence were assessed on a 0–6 scale, derealisation, gaps in awareness on a 0–4 scale, and PMERQ items on a 0–6 scale. For the reliability of repeated measures we provide within-person reliability estimates alpha (for multilevel estimates, also see Table S9 in the Supplementary Material). For the PMERQ we report coefficient omega.

Estimates for reliability coefficient omega were above 0.70 for most subscales in our sample (ranging from 0.74, 95% CI [0.60, 0.83] to 0.89, 95% CI [0.83, 0.93]; see ^32^). Only results regarding the *Expressive Suppression* subscale should be interpreted with caution using a reliability coefficient of 0.68 and a wide 95% CI [0.03, 0.80]. All analyses building on the PMERQ include data from 87 participants only, as one individual did not answer the questionnaire. Individual responses to items were missing on the following subscales: *Avoid Unpleasant Situations*, *Resolve Conflicts*, *Focus Elsewhere*, *Consider Benefits*, *Reduce Importance*, and *Expressive Suppression* (for a detailed overview of number of valid responses for each item and versions of the questionnaire and the specific items that they involved, please see Table S4 in the Supplementary Material; for handling of missing data refer to the statistical analysis section). All missing responses occurred because several PMERQ items in the German translation were being reformulated at that time (for documentation, see^16^).

### Assessments in daily life

After baseline diagnostics, participants used their smartphones to answer questions about their everyday experiences. Participants were given up to 12 audio prompts daily between 9:00 a.m. and 9:00 p.m. for 1 week, totalling 84 prompts. Four prompts were sent within 3 random 60-min intervals each day (morning, afternoon, evening). Participants were given up to 4 min to respond, ensuring evenly spaced time lags of approximately 15 min between prompts. A detailed description of the data collection process is available elsewhere^32^. An example schedule for our EMA assessments is provided in Figure S2, an overview of the times of realised prompts is available in Figure S3 in the Supplementary Material.

#### Momentary affective arousal and valence

We used items from the Multidimensional Mood Questionnaire (MDMQ;^45^) to assess momentary arousal and valence^46^. Participants reported affective arousal using two bipolar items (“relaxed-tense”/”entspannt-angespannt” and “agitated-calm”/”unruhig-ruhig”) and affective valence using two bipolar items (“content-discontent”/”zufrieden-unzufrieden” and “unwell-well”/”unwohl-wohl”). Responses were recorded on a scale ranging from 0 (*not at all*) to 6 (*very much*). For within-person internal consistency, alphas were estimated at 0.83, 95% CI [0.82, 0.83] for affective arousal and at 0.82, 95% CI [0.82, 0.83] for affective valence (also see Table S9 in the Supplementary Material for between- and multilevel reliability estimates).

#### Momentary dissociative states

Participants reported on current dissociation using two subscales from the brief DSS^41^(German items^40^). Derealisation was rated using two items (“At the moment, things around me seem strange or unreal.” and “I feel like I am in a movie—like nothing that is happening is real.”). Gaps in awareness were rated using two items as well (“At the moment, I realize I am not paying attention to what is going on around me.” and “At the moment, I am so focused on something going on in my mind that I lose track of what is happening around me.”). Responses were recorded on a scale ranging from 0 (*not at all)* to 4 (*very*). Prior studies have shown adequate within-person variability in these DSS-B items when used in ecological momentary assessments^9^. For the present research project, the DSS-B subscales were modified to inquire dissociative experiences in the current moment. For the within-person internal consistency, alphas were estimated at 0.83, 95% CI [0.82, 0.84] for derealisation and at 0.80, 95% CI [0.79, 0.81] for gaps in awareness (also see Table S9 in the Supplementary Material for between- and multilevel reliability estimates).

### Statistical analyses

We investigated the predictive links between habitual emotion regulation strategy use at baseline and daily affective arousal and valence and dissociation using Dynamic Structural Equation Modelling (DSEM^47^). The approach separates interindividual between-person differences (trait levels) from within-person fluctuations around this value and allows investigation of interindividual differences in autoregressive and cross-lagged associations. The DSEM is implemented within a Bayesian framework, with parameters estimated using a Markov Chain Monte Carlo procedure. All models included autocorrelations of order 1 (AR[1]) at the within-person level and used latent variables to account for measurement error when evaluating self-reported measures (see Table S5 in the Supplementary Material for our measurement models). Within-person standardised results were based on the M*plus* default procedure^48^. Varying time intervals between assessments were accounted for by inserting missing data for omitted prompts^47^. All models used the M*plus* default diffuse priors. In Bayesian analytic frameworks, priors represent prior information about model parameters; here, we employed weakly informative (“diffuse”) prior distributions, given that no prior model-specific information was available to inform the analysis (full prior distributions for all model parameters are available under https://doi.org/10.17605/OSF.IO/8X3QP). Two Markov chains were used, with a burn-in period of approximately half the minimum number of iterations (10,000) and thinning set to THIN = 10, such that every tenth iteration was retained. Convergence was assumed if the potential scale reduction factor fell below 1.10 for all parameters and after the inspection of trace plots, using at least 20,000 total iterations with the default Gibbs sampler for Markov Chain Monte Carlo. A summary of additional model convergence diagnostics is provided in the Supplementary Material, along with Tables S15 to S17. Although convergence was acceptable for all models, models including *Reduce Importance* as a predictor or predictive effects on cross-lagged associations as an outcome converged comparatively worse than the others, suggesting that these findings should be interpreted more cautiously.

For our hypothesis tests, we looked at the posterior medians and 95% credible intervals (CIs) based on the posterior distributions of the corresponding model estimates. Additionally, M*plus* provides a one-tailed posterior *p* value for each parameter, defined as the proportion of the posterior distribution on the opposite side of zero from the estimate itself, which is the proportion below zero for a positive estimate, or above zero for a negative estimate^49^. We doubled each of these one-tailed values to obtain the p values reported in Tables 2, 3, 4 and 5, which we then compared to a two-tailed α criterion of 0.05. We acknowledge that this approach, and the subsequent Holm correction, do not carry the same theoretical justification they would in a fully frequentist analysis. We included it nonetheless as small *p* values practically flag coefficients whose credible intervals likely do not cover zero^49^ and to include a conservative adjustment for interpreting a large number of coefficients.

**Table 2:** Habitual use of emotion regulation strategies predicting the means of affective arousal, affective valence, and dissociation in daily life (*N* = 87).

Prediction of Means																
Predictor	Arousal				Valence				Derealisation				Gaps in Awareness			
	Estimate	95% CI	*p*	*p* (adj.)	Estimate	95% CI	*p*	*p* (adj.)	Estimate	95% CI	*p*	*p* (adj.)	Estimate	95% CI	*p*	*p* (adj.)
Confront Unpleasant Situations	0.01	[-0.25, 0.26]	0.968	1.000	-0.06	[-0.30, 0.20]	0.664	1.000	-0.08	[-0.32, 0.16]	0.518	1.000	-0.07	[-0.31, 0.20]	0.614	1.000
Avoid Unpleasant Situations	-0.02	[-0.28, 0.25]	0.892	1.000	0.14	[-0.12, 0.39]	0.292	1.000	0.08	[-0.18, 0.33]	0.534	1.000	0.05	[-0.22, 0.31]	0.710	1.000
Resolve Conflicts	-0.15	[-0.39, 0.11]	0.252	1.000	0.10	[-0.15, 0.34]	0.438	1.000	-0.16	[-0.39, 0.09]	0.196	1.000	-0.18	[-0.42, 0.07]	0.154	1.000
Avoid Conflicts	0.02	[-0.24, 0.27]	0.894	1.000	-0.00	[-0.25, 0.25]	0.99	1.000	0.06	[-0.18, 0.29]	0.620	1.000	-0.04	[-0.28, 0.21]	0.786	1.000
Focus Elsewhere	-0.02	[-0.32, 0.29]	0.926	1.000	-0.09	[-0.38, 0.24]	0.598	1.000	-0.05	[-0.35, 0.26]	0.768	1.000	-0.10	[-0.39, 0.21]	0.540	1.000
Cognitively Distract	-0.03	[-0.29, 0.22]	0.806	1.000	-0.01	[-0.26, 0.25]	0.944	1.000	-0.05	[-0.30, 0.19]	0.670	1.000	-0.16	[-0.40, 0.10]	0.216	1.000
Consider Benefits	0.18	[-0.08, 0.42]	0.168	1.000	-0.10	[-0.34, 0.15]	0.436	1.000	0.04	[-0.21, 0.28]	0.750	1.000	-0.04	[-0.29, 0.22]	0.770	1.000
Reduce Importance	-0.01	[-0.35, 0.33]	0.978	1.000	-0.00	[-0.32, 0.32]	0.996	1.000	-0.02	[-0.37, 0.35]	0.934	1.000	0.06	[-0.36, 0.47]	0.780	1.000
Emotional Sharing	-0.06	[-0.31, 0.20]	0.648	1.000	0.18	[-0.08, 0.41]	0.172	1.000	**-0.25** ^**†**^	**[-0.47**,** -0.01]**	**0.042**	**1.000**	-0.19	[-0.42, 0.06]	0.124	1.000
Expressive Suppression	-0.08	[-0.51, 0.71]	0.748	1.000	-0.14	[-0.83, 0.33]	0.576	1.000	0.22	[-0.26, 0.82]	0.358	1.000	0.24	[-0.23, 0.83]	0.308	1.000

Note. No associations were statistically significant after applying the Holm correction for multiple comparisons (for details see S11 in the Supplementary Material). However, one significant uncorrected exploratory association is highlighted in bold. Mean values were determined as individual averages over all EMA measurements. Displayed are standardised Bayesian estimates. CI = credibility interval; *p* (adj.) = Holm-adjusted *p* value; ^**†**^*p* < .05 only at uncorrected α, not significant after Holm correction.

**Table 3:** Habitual use of emotion regulation strategies predicting the autoregressions of affective arousal, affective valence, and dissociation in daily life (*N* = 87).

Prediction of Autoregressions																
Predictor	Arousal_(t−1)_ → Arousal_(t)_				Valence_(t−1)_ → Valence_(t)_				Derealisation_(t−1)_ → Derealisation_(t)_				Gaps_(t−1)_ → Gaps_(t)_			
	Estimate	95% CI	*p*	*p* (adj.)	Estimate	95% CI	*p*	*p* (adj.)	Estimate	95% CI	*p*	*p* (adj.)	Estimate	95% CI	*p*	*p* (adj.)
Confront Unpleasant Situations	-0.15	[-0.42, 0.14]	0.298	1.000	-0.12	[-0.38, 0.16]	0.396	1.000	-0.18	[-0.44, 0.09]	0.184	1.000	-0.16	[-0.41, 0.11]	0.250	1.000
Avoid Unpleasant Situations	0.05	[-0.25, 0.33]	0.764	1.000	0.17	[-0.12, 0.44]	0.248	1.000	0.06	[-0.23, 0.33]	0.686	1.000	0.06	[-0.22, 0.33]	0.678	1.000
Resolve Conflicts	0.20	[-0.09, 0.46]	0.176	1.000	**0.30** ^**†**^	**[0.02**,** 0.54]**	**0.034**	**1.000**	0.03	[-0.24, 0.30]	0.832	1.000	-0.09	[-0.35, 0.18]	0.530	1.000
Avoid Conflicts	-0.13	[-0.40, 0.16]	0.378	1.000	0.07	[-0.20, 0.32]	0.610	1.000	-0.15	[-0.39, 0.12]	0.286	1.000	-0.17	[-0.41, 0.10]	0.216	1.000
Focus Elsewhere	0.09	[-0.30, 0.41]	0.636	1.000	0.10	[-0.22, 0.40]	0.536	1.000	-0.13	[-0.47, 0.20]	0.454	1.000	-0.06	[-0.42, 0.27]	0.740	1.000
Cognitively Distract	0.0	[-0.29, 0.28]	0.986	1.000	0.00	[-0.27, 0.27]	0.996	1.000	-0.06	[-0.33, 0.21]	0.660	1.000	-0.26	[-0.49, 0.01]	0.062	1.000
Consider Benefits	-0.02	[-0.29 0.26]	0.892	1.000	0.09	[-0.19, 0.35]	0.528	1.000	-0.11	[-0.36, 0.16]	0.440	1.000	-0.21	[-0.45, 0.05]	0.122	1.000
Reduce Importance	-0.22	[-0.57, 0.47]	0.472	1.000	0.15	[-0.53, 0.57]	0.794	1.000	-0.05	[-0.47, 0.44]	0.878	1.000	0.03	[-0.38, 0.50]	0.888	1.000
Emotional Sharing	-0.28	[-0.53, 0.00]	0.052	1.000	-0.12	[-0.38, 0.15]	0.388	1.000	-0.09	[-0.35, 0.19]	0.536	1.000	-0.27	[-0.50, 0.01]	0.058	1.000
Expressive Suppression	0.25	[-0.44, 0.72]	0.326	1.000	0.06	[-0.55, 0.59]	0.798	1.000	0.12	[-0.50, 0.67]	0.622	1.000	0.07	[-0.54, 0.59]	0.760	1.000

Note. No associations were statistically significant after applying the Holm correction for multiple comparisons (for details see S12 in the Supplementary Material). However, one significant uncorrected exploratory association is highlighted in bold. Displayed are standardised Bayesian estimates. CI = credibility interval; *p* (adj.) = Holm-adjusted *p* value; ^**†**^*p* < .05 only at uncorrected α, not significant after Holm correction.

**Table 4:** Habitual use of emotion regulation strategies predicting the contemporaneous cross-regression of affective arousal, affective valence, and dissociation in daily life (*N* = 87).

Prediction of Contemporaneous Cross-Regressions																
Predictor	Arousal_(t)_ ↔ Derealisation_(t)_				Arousal_(t)_ ↔ Gaps_(t)_				Valence_(t)_ ↔ Derealisation_(t)_				Valence_(t)_ ↔ Gaps_(t)_			
	Estimate	95% CI	*p*	*p* (adj.)	Estimate	95% CI	*p*	*p* (adj.)	Estimate	95% CI	*p*	*p* (adj.)	Estimate	95% CI	*p*	*p* (adj.)
Confront Unpleasant Situations	0.11	[-0.17, 0.37]	0.432	1.000	0.12	[-0.17, 0.38]	0.428	1.000	0.00	[-0.27, 0.27]	0.998	1.000	-0.04	[-0.32, 0.24]	0.770	1.000
Avoid Unpleasant Situations	-0.16	[-0.43, 0.14]	0.290	1.000	-0.25	[-0.51, 0.05]	0.102	1.000	-0.02	[-0.30, 0.27]	0.922	1.000	0.05	[-0.25, 0.34]	0.750	1.000
Resolve Conflicts	0.01	[-0.27, 0.29]	0.926	1.000	0.01	[-0.28, 0.28]	0.972	1.000	0.02	[-0.25 0.29]	0.892	1.000	-0.05	[-0.33, 0.24]	0.720	1.000
Avoid Conflicts	-0.13	[-0.39, 0.15]	0.370	1.000	-0.27	[-0.51, 0.01]	0.051	1.000	0.05	[-0.23, 0.31]	0.744	1.000	0.12	[-0.16, 0.38]	0.402	1.000
Focus Elsewhere	-0.12	[-0.43, 0.21]	0.472	1.000	0.02	[-0.29, 0.33]	0.902	1.000	0.16	[-0.16, 0.45]	0.338	1.000	-0.03	[-0.36, 0.29]	0.876	1.000
Cognitively Distract	0.01	[-0.27, 0.29]	0.930	1.000	-0.07	[-0.34, 0.21]	0.626	1.000	0.05	[-0.22, 0.32]	0.716	1.000	0.08	[-0.21, 0.37]	0.580	1.000
Consider Benefits	0.01	[-0.25, 0.28]	0.924	1.000	-0.03	[-0.31, 0.24]	0.812	1.000	0.06	[-0.21, 0.33]	0.662	1.000	0.07	[-0.22, 0.36]	0.624	1.000
Reduce Importance	0.04	[-0.71, 0.73]	0.944	1.000	-0.07	[-0.70, 0.67]	0.940	1.000	0.04	[-0.69, 0.70]	0.958	1.000	0.02	[-0.52, 0.55]	0.928	1.000
Emotional Sharing	0.16	[-0.13, 0.42]	0.286	1.000	0.09	[-0.19, 0.35]	0.530	1.000	0.01	[-0.26, 0.28]	0.952	1.000	-0.06	[-0.34, 0.22]	0.680	1.000
Expressive Suppression	0.06	[-0.56, 0.60]	0.834	1.000	0.15	[-0.51, 0.66]	0.644	1.000	0.06	[-0.53, 0.63]	0.848	1.000	0.17	[-0.47, 0.68]	0.594	1.000

Note. No associations were statistically significant after applying the Holm correction for multiple comparisons (for details see S13 in the Supplementary Material). Displayed are standardised Bayesian estimates. CI = credibility interval; *p* (adj.) = Holm-adjusted *p* value.

**Table 5:** Habitual use of emotion regulation strategies predicting the cross-lagged regressions of affective arousal, affective valence, and dissociation in daily life (*N* = 87).

Prediction of Cross-Lagged Regressions																
Predictor	Arousal_(t−1)_ → Derealisation_(t)_				Derealisation_(t−1)_ → Arousal_(t)_				Arousal_(t−1)_ → Gaps_(t)_				Gaps_(t−1)_ → Arousal_(t)_			
	Estimate	95% CI	*p*	*p* (adj.)	Estimate	95% CI	*p*	*p* (adj.)	Estimate	95% CI	*p*	*p* (adj.)	Estimate	95% CI	*p*	*p* (adj.)
Confront Unpleasant Situations	-0.04	[-0.40, 0.37]	0.850	1.000	-0.02	[-0.34, 0.30]	0.892	1.000	0.04	[-0.34, 0.38]	0.600	1.000	0.08	[-0.27, 0.41]	0.652	1.000
Avoid Unpleasant Situations	0.00	[-0.40, 0.37]	0.994	1.000	-0.04	[-0.38, 0.31]	0.844	1.000	-0.16	[-0.53, 0.24]	0.812	1.000	-0.05	[-0.40, 0.32]	0.430	1.000
Resolve Conflicts	-0.11	[-0.48, 0.28]	0.584	1.000	0.06	[-0.29, 0.43]	0.766	1.000	-0.21	[-0.53, 0.17]	0.262	1.000	0.04	[-0.29, 0.37]	0.860	1.000
Avoid Conflicts	-0.10	[-0.49, 0.28]	0.606	1.000	-0.04	[-0.36, 0.31]	0.802	1.000	-0.09	[-0.49, 0.34]	0.678	1.000	0.18	[-0.22, 0.56]	0.388	1.000
Focus Elsewhere	0.03	[-0.43, 0.52]	0.912	1.000	0.00	[-0.39, 0.42]	0.996	1.000	0.30	[-0.09, 0.63]	0.134	1.000	-0.22	[-0.52, 0.12]	0.206	1.000
Cognitively Distract	0.19	[-0.22, 0.55]	0.366	1.000	-0.15	[-0.46, 0.18]	0.358	1.000	-0.06	[-0.41, 0.29]	0.734	1.000	-0.06	[-0.36, 0.27]	0.718	1.000
Consider Benefits	-0.05	[-0.43, 0.36]	0.810	1.000	-0.04	[-0.36, 0.29]	0.828	1.000	0.00	[-0.35, 0.35]	0.986	1.000	-0.06	[-0.40, 0.28]	0.742	1.000
Reduce Importance	0.11	[-0.61, 0.67]	0.768	1.000	0.07	[-0.48, 0.58]	0.804	1.000	0.01	[-0.48, 0.52]	0.962	1.000	0.26	[-0.62, 0.71]	0.532	1.000
Emotional Sharing	0.31	[-0.07, 0.64]	0.106	1.000	-0.04	[-0.37, 0.31]	0.834	1.000	0.06	[-0.46, 0.54]	0.828	1.000	0.14	[-0.38, 0.60]	0.574	1.000
Expressive Suppression	0.07	[-0.55, 0.59]	0.982	1.000	-0.01	[-0.49, 0.49]	0.816	1.000	0.06	[-0.46, 0.54]	0.828	1.000	0.14	[-0.38, 0.60]	0.574	1.000

Predictor	Valence_(t−1)_ → Derealisation_(t)_				Derealisation_(t−1)_ → Valence_(t)_				Valence_(t−1)_ → Gaps_(t)_				Gaps_(t−1)_ → Valence_(t)_			
	Estimate	95% CI	*p*	*p* (adj.)	Estimate	95% CI	*p*	*p* (adj.)	Estimate	95% CI	*p*	*p* (adj.)	Estimate	95% CI	*p*	*p* (adj.)
Confront Unpleasant Situations	-0.08	[-0.43, 0.30]	0.648	1.000	0.00	[-0.36, 0.34]	0.992	1.000	0.03	[-0.39, 0.42]	0.888	1.000	0.00	[-0.37, 0.34]	0.996	1.000
Avoid Unpleasant Situations	0.01	[-0.35, 0.36]	0.962	1.000	0.05	[-0.33, 0. 43]	0.780	1.000	0.19	[-0.23, 0.57]	0.366	1.000	-0.12	[-0.47, 0.27]	0.548	1.000
Resolve Conflicts	0.05	[-0.32, 0.40]	0.806	1.000	-0.02	[-0.40, 0.33]	0.916	1.000	0.11	[-0.26, 0.47]	0.560	1.000	0.10	[0.24, 0.42]	0.586	1.000
Avoid Conflicts	0.00	[-0.34, 0.36]	0.996	1.000	0.03	[-0.32, 0.35]	0.888	1.000	0.23	[-0.14, 0.56]	0.862	1.000	-0.03	[-0.37, 0.30]	0.862	1.000
Focus Elsewhere	0.07	[-0.32, 0.43]	0.710	1.000	0.14	[-0.28, 0.52]	0.510	1.000	0.11	[-0.30, 0.47]	0.490	1.000	0.15	[-0.26, 0.53]	0.490	1.000
Cognitively Distract	0.08	[-0.30, 0.44]	0.690	1.000	0.02	[-0.35, 0.37]	0.904	1.000	-0.07	[-0.45, 0.34]	0.352	1.000	0.17	[-0.18, 0.50]	0.352	1.000
Consider Benefits	0.00	[-0.34, 0.35]	0.992	1.000	-0.01	[-0.34, 0.35]	0.980	1.000	0.02	[-0.40, 0.39]	0.934	1.000	-0.02	[-0.35, 0.31]	0.934	1.000
Reduce Importance	0.28	[-0.21, 0.70]	0.262	1.000	0.10	[-0.41, 0.58]	0.682	1.000	0.01	[-0.54, 0.54]	0.938	1.000	0.02	[-0.51, 0.53]	0.938	1.000
Emotional Sharing	-0.09	[-0.41, 0.26]	0.610	1.000	-0.05	[-0.44, 0.38]	0.798	1.000	0.02	[-0.34, 0.38]	0.926	1.000	0.19	[-0.15, 0.49]	0.284	1.000
Expressive Suppression	-0.09	[-0.56, 0.43]	0.748	1.000	0.03	[-0.52, 0.56]	0.924	1.000	0.13	[-0.59, 0.45]	0.652	1.000	-0.09	[-0.60, 0.48]	0.750	1.000

Note. No associations were statistically significant after applying the Holm correction for multiple comparisons (for details see S14 in the Supplementary Material). Displayed are standardised Bayesian estimates. CI = credibility interval; *p* (adj.) = Holm-adjusted *p* value.

Our analyses were performed in three steps. First, we ran univariate models to calculate latent means and autoregressions for affective arousal and valence, and dissociation variables (trait levels; Table 1) and then included the PMERQ subscales as between-person predictor variables to examine their relationships with these latent means and autoregressions (Tables 2 and 3). Second, we calculated bivariate models to investigate the autoregressive and contemporaneous within-person relations between affective arousal and valence, and dissociation in daily life (Table S6) and then included the PMERQ subscales as between-person predictor variables to examine their relationships with these contemporaneous relations (Table 4). Third, we included cross-lagged associations between affective arousal and valence, and dissociation variables at the within-person level (Table S10) and then included the PMERQ subscales as between-person predictor variables to examine their relationships with these cross-lagged relations (Table 5, also see Figure S4 in the Supplementary Material).

It is important to distinguish between three conceptually distinct sources of missingness in our data, only two of which are missing data in a conventional sense. The first type of missing data is not missing data in a conventional sense but was introduced deliberately to account for unequally spaced EMA assessments. Specifically, missing values were inserted at the 15-minute time points falling between the assessment blocks to account for varying time intervals between assessments, using the TINTERVAL function in M*plus* (see ^10,32^ for prior applications of this approach; see ^50^ for a more detailed explanation of the TINTERVAL procedure to handle these structurally missing values). Consequently, these missing data points do not overall completeness of EMA data but instead they are the result of a statistical modelling decision that enables the interpretation of effects over 15-minute intervals (see ^51^ for simulation study work supporting this approach).

The second type of missing data arose from participants not responding to the scheduled EMA prompts. Prior to our main analysis, we ran multilevel logistic regression models to determine whether exogenous variables (e.g., personality traits) predicted this second type of missing data. The results revealed that several baseline variables were significantly related to missingness (number of PTSD and depressive symptoms, conscientiousness). We used the AUXILIARY function in M*plus* to incorporate these variables into our analysis based on their association with missingness, thereby supporting a missing-at-random assumption and reducing bias in our estimates^52^ (auxiliary variables were modelled as correlates of the residuals of the observed PMERQ indicators rather than as predictors of the latent factors; for a detailed description of this approach please refer to ^53^).

The third type of missing data arose from missing responses on the several items of the German version of the PMERQ that was administered at baseline. Here, responses were missing because several items of the questionnaire were being revised (all revisions involved stylistic wording only, see ^16^ for details on the development of the German version of the PMERQ and Table S4 for details on which items contain missing values). As with missing values in the data from daily life, we ran multilevel logistic regression models to determine whether exogenous variables (e.g., personality traits) predicted this third type of missing data. The results revealed that several baseline variables were significantly related to missingness (number of BPD, PTSD, and depressive symptoms, openness, conscientiousness, extraversion, agreeableness, and neuroticism). We used the AUXILIARY function in M*plus* to incorporate these variables into our analysis based on their association with missingness. Although this approach tries to reduce the risk of bias introduced by missing data, residual measurement bias and loss of information cannot be fully ruled out for missing PMERQ responses. To ensure sufficient model fit of the PMERQ subscales including missing data, we allowed for correlated residuals in our factor models that were data-driven and not pre-specified (for details, also see S5).

Because we estimated separate models for each of the 10 emotion regulation strategies to predict the temporal associations of affective arousal and valence, and dissociation, we applied the Holm-Bonferroni step-down procedure to control for the family-wise error rate^54^. As shown in Tables S11 to S14 in the Supplementary Material, predictive effects for each type of parameter were treated as a separate family of tests. Predictive effects on within-person means, autoregressions and contemporaneous associations of affective arousal and valence, and dissociation comprised of 40 tests each, whereas predictive effects on cross-lagged associations enclosed 80 tests. For each family, all *p* values were ordered from smallest to largest and sequentially multiplied by (n − i + 1). Thus, the smallest *p* value in the cross-lagged family was multiplied by 80, the second-smallest by 79, and so forth. For our hypothesis tests we evaluated Holm-adjusted *p* values against a two-tailed α = 0.05. For exploratory purposes we also reported uncorrected *p* values.

All analyses were conducted using M*plus* version 9 (Mac)^43^ or the programming language R^55^ in the R Studio environment version 2024.12.1^56^. To automatically generate M*plus* scripts with all relevant predictors for each model and parse result outputs, we used the programming language Python version 3.13.2^57^ in the Spyder environment version 6.0.7^58^. All statistical scripts and additional resources are available under: https://doi.org/10.17605/OSF.IO/8X3QP.

## Results

### Preliminary analyses

Table 1 shows descriptive statistics for all measures. All contrasts reported in this section were tested as latent-variable contrasts. Overall, participants reported using disengagement-oriented emotion regulation strategies as often as engagement-oriented emotion regulation strategies (*M*_*Engagement*_ - *M*_*Disengagement*_ = -0.27, 95% CI [-0.55, 0.02], *z* = -1.83, *p* = .067). In addition, participants on average used strategies during the first three stages (situation selection, situation modification, attentional deployment) more often than they used strategies during the last two stages (cognitive change, response modulation): *M*_*Early*_ - *M*_*Late*_ = 1.74, 95% CI [1.44, 2.04], *z* = 11.42, *p* < .001. The disengagement-oriented strategy to *Avoid Unpleasant Situations* (*M* = 4.31, 95% CI [3.74, 4.89]) was the most frequently endorsed strategy, whereas the engagement-oriented strategy *Focus Elsewhere* (*M* = 2.86, 95% CI [2.50, 3.21]) was the least frequently endorsed strategy. Participants were significantly more inclined to *Avoid Unpleasant Situations* (*M* = 4.31, 95% CI [3.74, 4.89]) than to *Confront* them (*M* = 3.31, 95% CI [2.97, 3.65], *difference* = -1.00, *z* = -2.81, *p* = .005), and to *Resolve Conflicts* (*M* = 4.24, 95% CI [3.92, 4.55]) rather than to *Avoid* them (*M* = 3.36, 95% CI [2.97, 4.00], *difference* = 0.88, *z* = 2.60 *p* = .009). They chose to *Focus Elsewhere* (*M* = 2.86, 95% CI [2.50, 3.21]) less than to *Cognitively Distract* (*M* = 4.27, 95% CI [3.97, 4.58], *difference* = -0.40, *z* = 5.95 *p* < .001) and to *Consider Benefits* (*M* = 2.97, 95% CI [2.66, 3.27]) less than to *Reduce Importance* (*M* = 3.26, 95% CI [2.83, 3.68], *difference* = -0.29, *z* = -1.59 *p* = .111). Finally, participants chose *Emotional Sharing* (*M* = 3.45, 95% CI [3.13, 3.77]) as often as *Expressive Suppression* (*M* = 4.27, 95% CI [3.67, 4.88], *difference* = − 0.0,82, *z* = 1.89 *p* = .056).

The average number of answered prompts in daily life was 75.94 out of 84 (90.41%, *SD* = 8.96). Because ratings were mandatory, participants answered all affective arousal and valence, and dissociation items if they responded to the prompts. The results in Table 1 show that our sample reported levels around the mid-point of the scale for momentary affective arousal (*M* = 3.06, 95% CI [2.89, 3.23]) and valence (*M* = 2.87, 95% CI [2.68, 3.07]). The *ICC*s were estimated at 0.27 (95% CI [0.18, 0.39]) for arousal and 0.39 (95% CI [0.28, 0.53]) for valence, demonstrating sufficient variability on the within-level. Participants showed mean levels for derealisation (*M* = 1.15, 95% CI [1.02, 1.29]) and gaps in awareness (*M* = 1.07, 95% CI [0.94, 1.21]) in daily life that match those of clinical populations with moderate to high levels of dissociation^40^. The *ICCs* were estimated at 0.41 (95% CI [0.30, 0.54]) for derealisation and 0.42 (95% CI [0.27, 0.56]) for gaps in awareness, demonstrating sufficient variability at the within-level.

### Emotion regulation predicting daily affective arousal, affective valence, and dissociation

As shown in Table 2 affective arousal was not predicted by engagement-oriented subscales (standardised estimates ranging between − 0.15 and 0.18), or disengagement-oriented subscales (standardised estimates in a range between − 0.08 and 0.02). Similarly, affective valence was not predicted by engagement-oriented subscales (standardised estimates ranging between − 0.10 and 0.18), or disengagement-oriented subscales (standardised estimates in a range between − 0.14 and 0.14). For *p* values before and after applying the Holm correction for multiple testing, please refer to Table S11 in the Supplementary Material.

Additionally, as displayed in Table 3, autoregressive effects for affective arousal were neither predicted by engagement-oriented (standardised estimates ranging between − 0.28 and 0.20), nor disengagement-oriented strategies (standardised estimates in a range between − 0.22 and 0.25). Similarly, most autoregressive effects for affective valence were not predicted by engagement-oriented (standardised estimates ranging between − 0.12 and 0.30), or disengagement-oriented strategies (standardised estimates in a range between 0.00 and 0.17). However, as reported in Table 3, there was one exception. The engagement-oriented strategy from the situational modification stage, *Resolve Conflicts*, significantly predicted high autoregressive parameters (i.e., more temporal stability) in affective valence (*γ* = 0.30, *p* = .034, 95% CI [0.02, 0.54]). While the uncorrected credibility interval did not include zero, the predictive effect of *Resolve Conflicts* on the temporal stability of valence was no longer significant after applying the Holm correction for multiple testing (refer to Table S12 in the Supplementary Material for corrected and uncorrected *p* values).

Finally, as shown in Table 2, the PMERQ subscales did not significantly predict most latent means of dissociation. Standardised estimates for engagement-oriented strategies predicting derealisation ranged between − 0.25 and 0.04, and predictive effects for disengagement-oriented strategies ranged between to -0.05 and 0.22. Again, there was one exception as displayed in Table 2. The engagement-oriented strategy from the response modulation stage, *Emotional Sharing*, significantly predicted low mean levels of derealisation (*γ* = -0.25, *p* = .042, 95% CI [-0.47, -0.01]). However, after the Holm correction for alpha cumulation was applied, this effect did not remain significant (see Table S11 in the Supplementary Material for corrected and uncorrected *p* values). Standardised effects for engagement-oriented strategies predicting gaps in awareness ranged between − 0.19 and − 0.04, and predictive effects for disengagement-oriented strategies ranged between − 0.16 and 0.24.

Table 3 further demonstrates that neither engagement-oriented subscales predicted autoregressive effects of derealisation (standardised estimates ranging between − 0.18 and 0.03), nor disengagement-oriented strategies (standardised estimates in a range between − 0.15 and 0.12). Estimates for engagement-oriented strategies predicting gaps in awareness ranged between − 0.27 and − 0.06, and estimates for disengagement-oriented strategies predicting gaps in awareness ranged between − 0.26 and 0.07 (see Table S12 in the Supplementary Material for corrected and uncorrected *p* values).

### Emotion regulation predicting dynamics between affective arousal, affective valence, and dissociation

As displayed in Table 4, the habitual use of emotion regulation strategies did not significantly predict the contemporaneous associations between affective arousal and valence, and dissociation (see Table S13 in the Supplementary Material for corrected and uncorrected *p* values). Standardised effect sizes between affective arousal and derealisation fell between − 0.12 and 0.16 for engagement-oriented strategies, and between − 0.16 and 0.06 for disengagement-oriented strategies. For the link between affective valence and derealisation, they ranged between 0.00 and 0.16 for engagement-oriented strategies, and between − 0.02 and 0.06 for disengagement-oriented strategies. For the relation between affective arousal and gaps in awareness, they ranged between − 0.03 and 0.14 for engagement-oriented strategies, and between − 0.27 and 0.15 for disengagement-oriented strategies. For the association between affective valence and gaps in awareness, estimates ranged between − 0.06 and 0.07 for engagement- oriented strategies, and between 0.02 and 0.17 for disengagement-oriented strategies.

In addition, Table 5 shows that the habitual use of emotion regulation did not significantly predict lagged associations between affective arousal and valence, and dissociation (see Table S14 in the Supplementary Material for corrected and uncorrected *p* values). For the link between arousal and later derealisation, standardised estimates ranged between − 0.11 and 0.31 for engagement-oriented strategies and between − 0.10 and 0.19 for disengagement-oriented strategies. For the link between arousal and later gaps in awareness, estimates ranged between − 0.21 and 0.30 for engagement-oriented strategies, and between − 0.16 and 0.06 for disengagement-oriented strategies. For the link between valence and later derealisation, estimates ranged between − 0.09 and 0.07 for engagement-oriented strategies, and between − 0.09 and 0.28 for disengagement-oriented strategies. For the link between valence and later gaps in awareness, estimates ranged between 0.02 and 0.11 for engagement-oriented strategies, and between − 0.13 and 0.23 for disengagement-oriented strategies. For the link between derealisation and later arousal, standardised estimates ranged between − 0.04 and 0.06 for engagement-oriented strategies, and between − 0.15 and 0.07 for disengagement-oriented strategies. For the link between derealisation and later valence, estimates ranged between − 0.02 and 0.14 for engagement-oriented strategies, and between 0.02 and 0.10 for disengagement-oriented strategies. For the link between gaps in awareness and later arousal, estimates ranged between − 0.22 and 0.14 for engagement-oriented strategies, and between − 0.06 and 0.26 for disengagement-oriented strategies. Ultimately, for the link between gaps in awareness and later valence, estimates ranged between − 0.02 and 0.19 for engagement-oriented strategies, and between − 0.12 and 0.17 for disengagement-oriented strategies.

## Discussion

This study explored how the habitual use of engagement-oriented and disengagement-oriented emotion regulation strategies, as assessed by the PMERQ, predicts affective arousal and valence, and dissociation in the daily lives of individuals with PTSD, BPD, and DD. Although our sample reported frequent habitual use of disengagement-oriented strategies, contrary to our expectations, scores on the PMERQ subscales did not robustly predict mean levels of affective arousal, or valence, or dissociation, or their temporal dynamics, in our sample under the present analytic approach after correcting for multiple comparisons. However, the absence of statistically robust associations should not be interpreted as evidence of equivalence or as proof that meaningful relationships are absent due to limited sensitivity to small to moderately small effect sizes.

Still, we identified two significant effects before correcting for multiple testing that are treated as uncorrected exploratory associations. In the following sections, we discuss theoretical implications of our null results and consider potential methodological explanations.

### Implications and possible explanations for null results

Prior meta-analytic work has documented robust associations between emotion regulation difficulties and affective arousal and negative valence, and dissociation^26–28^. Inconsistent with these meta-analytic findings, our intensive longitudinal design showed no robust associations of habitual emotion regulation predicting daily affective arousal and valence, and dissociation. The absence of robust effects in the present study should not be taken as general evidence that habitual emotion regulation is unrelated to daily psychopathology. Rather, several design- and sample-specific explanations merit consideration. It is worth noting that our design cannot reliably distinguish between these possibilities.

First, our between-person sample size of *N* = 87 for predictive associations based on the PMERQ may have limited statistical power, reducing our ability to detect small to moderately small effects. Thus, the absence of medium-to-large-sized predictive effects should not be taken as evidence against smaller associations, which cannot be ruled out and should be examined in larger, adequately powered longitudinal studies. Relatedly, the limited between-person sample size, likely also contributed to model convergence being acceptable overall, yet comparatively worse for models including predictive effects on cross-lagged associations, limiting the confidence in these specific findings. Also, we chose to investigate a naturalistic clinical sample to ensure sufficient dissociation in daily life. This choice may have included individuals with heterogeneous diagnoses and pharmacological as well as psychotherapeutic treatments. Also, we chose to investigate a naturalistic clinical sample to ensure sufficient levels of dissociation in daily life. This approach may have resulted in a heterogeneous sample with respect to diagnoses, as well as pharmacological and psychotherapeutic treatments. Moreover, the restricted range of dissociation in this sample may have attenuated associations with the predictor variables.

A second concern relates to measurement problems and missing responses due to item reformulations in the PMERQ scales used as predictors for habitual emotion regulation. Most affected by this were the *Reduce Importance* and *Expressive Suppression* subscales showing a high proportion of missing responses due to these reformulations, which resulted in less stable model estimation and lower reliability (for more details refer to the limitations and Tables S4, S5 and S15 to S17 in the Supplementary Material).

Third, the dense sampling plan of our EMA design, aimed to capture dynamics in daily life, may have limited our ability to detect predictive associations that unfold over longer periods. A recent longitudinal study in a community sample found that habitual use of adaptive affect regulation strategies predicted low levels of dissociation and habitual use of maladaptive strategies predicted high levels of dissociation at longer time scales^33^. In this study, two measurements were spaced 2 months apart, in marked contrast to our intensive longitudinal approach. Therefore, habitual measures of affect regulation may be ecologically valid to predict dissociation over weeks or months rather than minutes or hours.

Fourth, we used a habitual measure of emotion regulation to predict outcomes in daily life. This approach raises the possibilities of contextual moderation and a mismatch between trait-level self-report measures and the momentary regulatory processes that unfold in daily life. Recent work has questioned the ecological validity of trait-level self-report measures of emotion regulation^59,60^. Some authors argue that habitual self-reports may reflect a bias toward perceived regulatory needs and their emotional consequences rather than actual in-the-moment behaviour^59^. Consistent with the argument that habitual and momentary emotion regulation measures may reflect distinct facets of the same construct, Weiss and colleagues found in a recent longitudinal study that habitual emotion regulation difficulties predicted greater variability in momentary emotion regulation^61^. Momentary emotion regulation difficulties, in turn, predicted more negative momentary affective valence, suggesting that habitual and momentary assessments capture meaningfully different aspects of the regulatory process. Notably, a handful of studies in the Boemo et al. (2022) meta-analysis examined state regulation in relation to state-level rather than trait-level habits^28–30^, suggesting that state- measures of emotion regulation may offer more ecologically valid predictive signals for affective arousal and valence in daily life than habitual instruments.

Fifth, our assessment of habitual emotion regulation was grounded in the widely used process model of emotion regulation^2,3^, which emphasises hierarchical cognitive mechanisms as central to emotion regulation. However, a recent systematic review notes that alternative frameworks capture facets of emotion regulation that receive less attention within this cognitive-dominant perspective^12^. For instance, the difficulties in emotion regulation model encompasses very broad dimensions, such as awareness, understanding, and acceptance of emotions, as well as the ability to act in accordance with personal goals despite emotional distress^62^. Regulatory flexibility models further propose that adaptive emotion regulation is not defined by the use of specific strategies, but by the capacity to flexibly adapt regulatory responses to situational demands and personal resources^63^. Converging evidence similarly underscores that the effectiveness of any given strategy may depend on contextual and individual factors rather than fixed strategy properties^64^. Nevertheless, Bruno et al.’s (2025) meta-analysis reported consistent, medium-sized effects across theoretical perspectives of emotion regulation and their links to dissociation, which renders this explanation comparatively less plausible^26^.

Altogether, our null findings do not support a true absence of effects of habitual emotion regulation on daily psychopathology in general. Clinically, these findings do not provide evidence regarding the efficacy of targeting specific emotion-regulation strategies in treatment. Future intervention studies are needed to determine whether modifying specific emotion-regulation strategies produces clinically meaningful changes in these outcomes.

### Descriptive uncorrected associations

Although we did not observe robust effects of the habitual use of emotion regulation strategies on affective arousal and valence, and dissociation, we identified two uncorrected associations: First, frequent use of *Emotional Sharing*, which is an engagement-oriented strategy from the response modulation stage, predicted low mean levels of derealisation. Second, more frequent use of *Resolve Conflicts*, an engagement-oriented strategy from the situation modification stage, predicted high temporal stability of affective valence, which is not intrinsically beneficial or detrimental as both positive and negative affects last longer. Taken together, both associations point to possible exploratory hypotheses for future confirmatory research whereas none of them remained statistically robust after correction for multiple testing.

### Limitations and future research

As a novel contribution, this study provides a fine-grained examination of 10 individual emotion regulation strategies and their roles as predictors of affective arousal and valence, and dissociation in daily life in our data set. We focused on a well-characterised clinical sample comprised of individuals with PTSD, BPD, and DD and high levels of dissociation. However, our study also had several limitations.

A first group of limitations concerns our sample. Simulation analyses suggest that our sample size may be insufficient to distinguish small to moderately small effects from zero. Additional convergence diagnostics imply acceptable, yet comparatively less stable convergence for the complex models including predictive effects on cross-lagged associations. Future studies should prioritise larger samples. Ideally, such studies also adopt a confirmatory approach with pre-registered hypotheses. Additionally, elevated dissociation may have restricted variance in the outcomes and attenuated associations with PMERQ predictors. Also, the high proportion of female participants in our sample (76.10%) aligns with the typical gender distribution in clinical populations with dissociative symptoms^19^; however, this bias limits generalisability. Furthermore, our sample was relatively young. Prior research suggests that the link between emotion regulation and dissociation may be stronger in older adults (*β* = 0.14 – 0.21), who typically exhibit less flexibility in emotion regulation strategy use than younger adults^26^. The clinical context also restricted the range of dissociation and involved heterogeneous pharmacological and psychotherapeutic treatment (more details are available in Tables S1 and S2 in the Supplementary Material). Future research should, thus, consider demographically more diverse samples.

Second, as elaborated upon above, we used a habitual emotion regulation measure to predict daily affective arousal and valence, and dissociation. Yet, habitual (trait) and momentary (state) assessments capture related but distinct aspects of emotion regulation^65^. Global habitual measures may reflect more the perceived need to regulate emotions and their emotional consequences, rather than actual daily regulation patterns^58^. To capture the flexible, situational nature of emotion regulation processes^66^, future studies should consider momentary assessments of emotion regulation, as in^29^ and^30^. Such daily measures can assess situational goals and reveal individual patterns of affect regulation, enabling tailored interventions^67^. Future research may yield a more ecologically valid image of emotion regulation by further investigating momentary and multidimensional assessment methods^61,68^.

A further limitation concerns missing responses and poor model fit for several PMERQ subscales (i.e., our predictor variables). Although missing items due to reformulations where individuals did not have the opportunity to answer these items were addressed by including AUXILIARY variables within our statistical framework to reduce the risk of bias, residual measurement bias and loss of information remain possible. Parameter estimates may have been biased for several PMERQ subscales with a small number of observations such as the *Reduce Importance* subscale, which showed comparatively less stable model convergence, and the *Expressive Suppression* subscale, which displayed limited reliability, warranting a cautious interpretation of findings involving these scales. Allowing for correlated residuals in a data-driven way may have posed the risk of sample-specific overfitting. Future work should further examine the validity of PMERQ scores in clinical populations.

Fourth, we included several baseline characteristics associated with missingness, such as the number of BPD, PTSD, and depressive symptoms, as auxiliary variables in our analyses, making the MAR assumption more plausible. Nevertheless, we cannot rule out situational reasons for non-response, such as acute distress, fatigue, lack of sleep, interpersonal conflict, or strong situational demands. Given the response rate of over 90%, however, we consider this a minor concern.

Fifth, additional emotion-related processes, such as alexithymia, warrant further investigation. Alexithymia involves difficulties in identifying, describing, and differentiating emotions, as well as a tendency toward externally oriented thinking^69^. As a stable, transdiagnostic risk factor for psychopathology including dissociation (*r* = .30;^26, 70, 71^), alexithymia may limit emotional flexibility and the appropriate selection of regulation strategies^72^. Future studies should consider incorporating such underlying mechanisms.

Finally, the present study assessed only two facets of dissociation, namely derealisation and gaps in awareness, and it is possible that the observed patterns do not generalise to other dissociative experiences^22, 73^. In addition, operationalising dissociation through gaps in awareness may lead to overlap with related constructs, such as mind-wandering or flow, which are not necessarily maladaptive and may be less strongly linked to emotion regulation. Future research should use more focused conceptualisations of dissociation^74^. Moreover, despite our rationale to include individuals with BPD, PTSD, and DD, data from all participants were analysed as a pooled group. Exploratory comparisons did not indicate major differences between diagnostic groups (refer to Table S8). However, as our study only included four individuals with depersonalisation/derealisation disorder, the small subgroup size precludes meaningful conclusions regarding diagnostic differences and limits the generalisability of our fundings to this group as well as other diagnostic groups who experience dissociation. Moreover, diagnostic groups may have differed in the phenomenology of dissociation. Future research should examine a broader range of dissociative facets to determine whether the present findings are specific to the dimensions assessed here or reflect a more general underlying relationship.

## Conclusions

This study examined how the habitual use of 10 different emotion regulation strategies predicts subsequent daily levels of affective arousal and valence, and dissociation in a clinical sample. When applying strict statistical criteria, our intensive longitudinal data analyses found no robust associations between habitual emotion regulation strategy use and daily-life levels of affective arousal and valence, and dissociation in our sample; associations with temporal dynamics across affective arousal and valence, and dissociation were also non-significant. However, the absence of statistically robust associations should not be interpreted as proof that meaningful relationships are absent. Equally possible explanations include limited between-person sample size, resulting in limited statistical sensitivity to detect small to moderately small effect sizes and less stable convergence for models involving cross-lagged effects, specific sample characteristics, measurement problems and missing responses particularly affecting the *Reduce Importance* *and* *Expressive Suppression* subscales of the PMERQ, the context-dependence of the relationship between emotion regulation and daily psychopathology (i.e. a mismatch between state- and trait-level emotion regulation). Two descriptive uncorrected associations were observed that we suggest should be further investigated in the future: *Emotional Sharing* was linked to low mean levels of derealisation, whereas *Resolve Conflicts* was linked to high autoregressive parameters (i.e. temporal persistence) of affective valence. Both associations represent descriptive uncorrected findings and did not remain significant after correction for multiple testing. In summary, future research should further disentangle the impact of state and trait domains of emotion regulation on psychopathology and include momentary assessments of emotion regulation strategy use.

## Supplementary Information

Below is the link to the electronic supplementary material.

Supplementary Material 1

## Data Availability

Statistical code and additional resources are available at https://doi.org/10.17605/OSF.IO/8X3QP (including creation of latent variables, insertion of structural missing values, inclusion of auxiliary variables, DSEM estimation, extraction of posterior summaries, multiplicity correction, and parsing of model outputs into Excel files). After an initial embargo period reserved for use by students ending 01/01/2028, data will be made available at https://doi.org/10.5281/zenodo.7568590 and can be used without restrictions. If researchers wish to access the data sooner, they may contact J.B.H. directly. An R script was used to convert the original uploads into the right format for Mplus, which was uploaded to the OSF repository.
