# Supplementary material for: Emotion regulation strategy use and daily levels of affective arousal, affective valence and dissociation in a clinical sample
Source: Sci Rep. 2026 Sep 15;16:28774. doi: 10.1038/s41598-026-70906-7 (PMC13578288; doi:10.1038/s41598-026-70906-7)
Supplement: Supplementary file 1 [file 41598_2026_70906_MOESM1_ESM.pdf]

Supplementary Material

Figure S1 The Process Model of Emotion Regulation (Gross, 2015) with Specific Strategies From the According Questionnaire (PMERQ, Olderbak et al., 2022) 3

Figure S2 Example for the Experience Sampling Schedule During One Day 3

Figure S3 Distribution of Realised and Missing EMA Prompts Across the Day (N=88) 4

Figure S4 Within-person Model for Cross-lagged Dynamic Structural Equation Models..... 5

Table S1 Sample Characteristics (N=88) 6

Table S2 Main and Comorbid Mental Disorders (N=88) 7

Table S3 Data Collection in N = 88 Individuals with Severe Dissociative Symptoms 8

Table S4 Number and Percentage of Valid Responses on the Process Model of Emotion Regulation Questionnaire (Heekerens et al., 2024; N = 87) 9

Table S5 Confirmatory Factor Analysis Measurement Models for the PMERQ (Heekerens et al., 2024; N=87) 10

Table S6 Contemporaneous Associations Between Affective Arousal, Affective Valence, and Dissociative States in Daily Life and Habitual Use of Emotion Regulation Strategies at Baseline (N=87) 11

Table S7 Comparison of Contemporaneous Interrelations between PMERQ Scales (Heekerens et al., 2024; N=87): Latent Minus Manifest Estimates 12

Table S8 Comparison of Habitual Use of Emotion Regulation Strategies Predicting Means and Autoregressions of Affective Arousal, Affective Valence, and Dissociation in Daily Life for Participants with BPD (N=63) and PTSD (N=46) 13

Table S9 Multilevel Reliability Coefficients Alpha for Affective Arousal, Affective Valence and Dissociation in Daily Life on Within- and Between-person Level and for the Overall Composite Score (N=88) 14

Table S10 Results of the Temporal Associations Between Affective Arousal, Affective Valence, and Dissociation from Heekerens, Gross et al. (2026) 15

Table S11 Holm Correction for Test Family Prediction of Means (N = 40 Tests) 16

Table S12 Holm Correction for Test Family Prediction of Autoregressions (N = 40 Tests).. 16

Table S13 Holm Correction for Test Family Prediction of Contemporaneous Associations (N = 40 Tests) 17

Table S14 Holm Correction for Test Family Prediction of Cross-Lags (N = 80 Tests).......... 18

Table S15 Summary of Converge Parameters for Models Predicting Means and Autoregressions 20

Table S16 Summary of Converge Parameters for Models Predicting Contemporaneous Cross-regressions 20

Table S17 Summary of Converge Parameters for Models Predicting Cross-lagged Regressions 21

Summary of Model Convergence Diagnostics 19

References 22

Figure S1

The Process Model of Emotion Regulation (Gross, 2015) with Specific Strategies From the According Questionnaire (PMERQ, Olderbak et al., 2022)

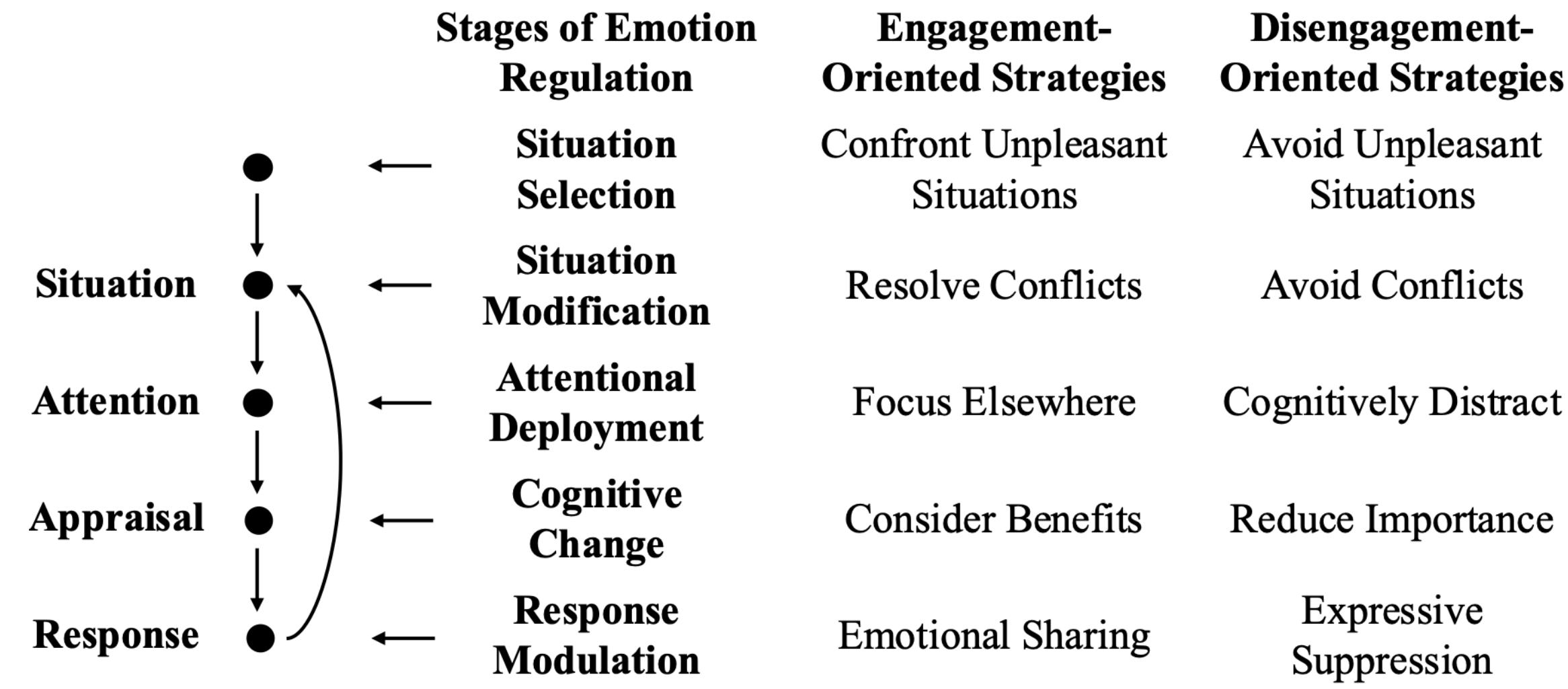

Note. The process model distinguishes ten strategies along the five stages of the emotion-generative process, as well as engagement- and disengagement orientations.

Figure S2

Example for the Experience Sampling Schedule During One Day

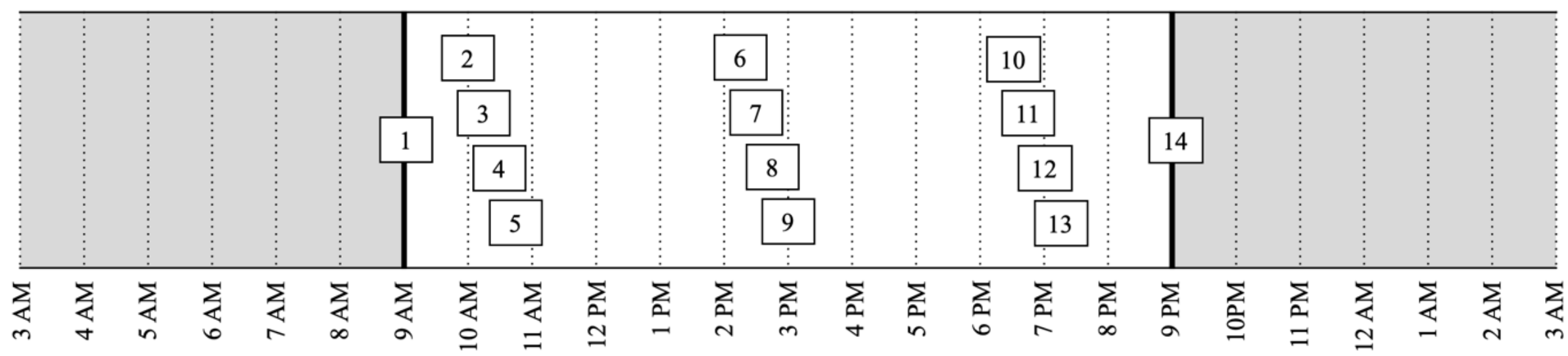

Note. Each box represents one prompt from the app.

Figure S3

Distribution of Realised and Missing EMA Prompts Across the Day

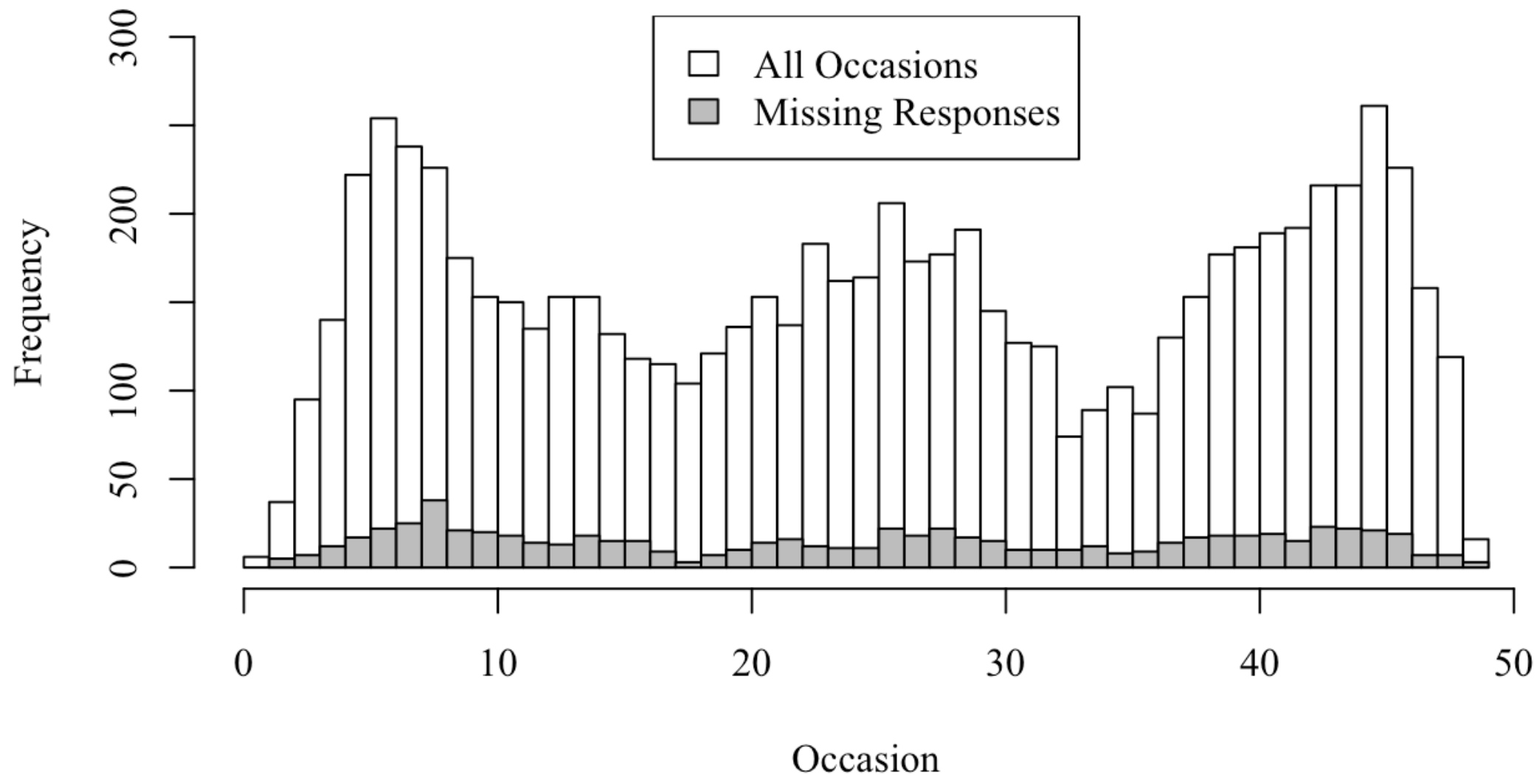

Note. In our discrete time grid occasion 0 stands for 9:00 AM, occasion 1 for 9:15 AM and so forth until 9:00 PM. The majority of inter-prompt intervals was consistent with our 15-minute time grid. Longer intervals emerged between assessment blocks, particularly overnight. The minimum interval length was 1, the maximum interval length was 88. The median interval length was 1, the interquartile range was 1.

Figure S4

Within-person Model for our Cross-lagged Dynamic Structural Equation Models (DSEM)

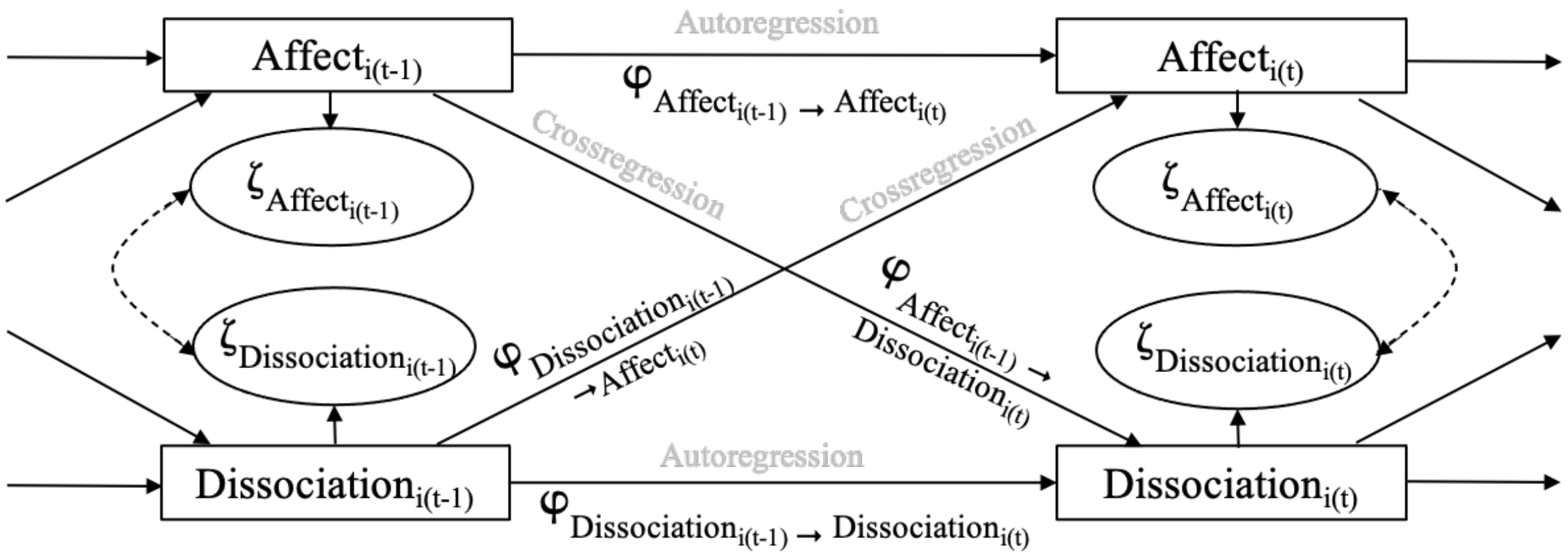

Note. Current dissociation was regressed on dissociation 15 minutes earlier (first-order autoregressive effect) and affective arousal/valence 15 minutes earlier (first-order cross-lagged effect). These relationships are modelled as random effects (φ). The models also take into account systematic changes in the dynamic variables (innovations) that the other variables in the model cannot explain (random innovation variances ζ).

Table S1

Sample Characteristics (N=88)

	N	%	Mean	SD	Range
**Age**	-	-	30.3	8.5	19-53
**Gender**					
Female	67	76.1	-	-	-
Male	10	11.4	-	-	-
Non-binary	9	10.2	-	-	-
Not specified	1	1.1	-	-	-
Other	1	1.1	-	-	-
**Sex**					
Female	78	88.6	-	-	-
Male	10	11.4	-	-	-
**Relationship status**					
Single	64	72.7	-	-	-
Committed Relationship, Not Living with Partner	8	9.1	-	-	-
Committed Relationship, Living with Partner	6	6.8	-	-	-
Married, Not Living with Partner	1	1.1	-	-	-
Married, Living with Partner	5	5.7	-	-	-
Divorced	4	4.5	-	-	-
**Ethnicity**					
White	73	83.0	-	-	-
Arab/Turk	3	3.4	-	-	-
Asian	1	1.1	-	-	-
Black	1	1.1	-	-	-
Mixed race	1	8.0	-	-	-
Not specified	3	3.4	-	-	-
**Education**					
Secondary School Leaving Certificate	3	3.4	-	-	-
Intermediate School Leaving Certificate	24	27.3	-	-	-
Advanced Technical College Entrance Qualification	16	18.2	-	-	-
University Entrance Qualification	45	51.1	-	-	-
**Employment**					
Employed Full-Time	23	26.1	-	-	-
Employed Half-Time	16	18.2	-	-	-
Occasionally, Marginally, or Irregularly Employed	8	9.1	-	-	-
Not Employed	41	46.6	-	-	-
**Current Outpatient Psychotherapy**	47	53.4%	-	-	-
**Current Psychopharmacotherapy**					
Antidepressant (e.g., SSRI, SSNRI)	44	50.0%	-	-	-
Antipsychotic	19	21.6%	-	-	-
Anxiolytic	8	9.1%	-	-	-
Stimulant (Methylphenidate)	5	5.7%	-	-	-
Other	6	6.8	-	-	-

Note. This table is derived from Heekerens, Gross, et al. (2026), with kind permission to be reused for this study. SD = standard deviation; SSRI = selective reuptake inhibitor; SSNRI = selective serotonin norepinephrine reuptake inhibitor.

Table S2

Main and Comorbid Mental Disorders (N=88)

	N	%
**Main Diagnosis (DSM-5)**		
Borderline Personality Disorder (301.83)	63	71.59
Post-traumatic Stress Disorder (309.81)	46	52.27
Depersonalisation/Derealisation Disorder (300.6)	4	4.55
**Comorbid Diagnosis (DSM-5)**		
Amphetamine (or other stimulant)-induced bipolar and related disorder (292.84)	1	1.14
Major depressive disorder, Single episode, Unspecified (296.20)	1	1.14
Single depressive episode, current episode mild (296.21)	1	1.14
Single depressive episode, current episode moderate (296.22)	2	2.27
Single depressive episode, currently in remission (296.26)		7.95
Recurrent depressive disorder, current episode mild (296.31)	2	2.27
Recurrent depressive disorder, current episode moderate (296.32)	11	12.50
Recurrent depressive disorder, current episode mild (296.33)	2	2.27
Recurrent depressive disorder, currently in remission (296.36)	36	40.91
Bipolar I disorder, Current or most recent episode Depressed, In full remission (296.56)	1	1.14
Panic disorder (300.01)	1	1.14
Generalized anxiety disorder (300.02)	2	2.27
Agoraphobia (300.22)	3	3.41
Social anxiety disorder (300.23)	7	7.95
Obsessive-compulsive disorder (300.3)	4	4.55
Persistent depressive disorder (dysthymia) (300.4)	5	5.68
Narcissistic personality disorder (301.81)	1	1.14
Alcohol use disorder, current severity moderate (303.90)	4	4.55
Alcohol use disorder, current severity mild (305.00)	8	9.09
Cannabis use disorder, current severity mild (305.20)	6	6.82
Sedative, hypnotic, or anxiolytic use disorder, current severity mild (305.70)	3	3.41
Opioid use disorder, current severity mild (305.70)	1	1.14
Eating disorder, unspecified (307.50)	2	2.27
Bulimia nervosa (307.51)	2	2.27
Attention-deficit/hyperactivity disorder, combined presentation (314.01)	2	2.27
**Diagnostic Features (DSM-5)**		
Depersonalisation	73	82.95%
Derealisation	56	63.64%
Disruption of Identity	6	6.82%
Memory Problems	30	34.09%

Note. This table is derived from Heekerens, Gross, et al. (2026), with kind permission to be reused for this study. SD = standard deviation; SSRI = selective reuptake inhibitor; SSNRI = selective serotonin norepinephrine reuptake inhibitor.

Table S3

Data Collection in N = 88 Individuals with Severe Dissociative Symptoms

Baseline Diagnostics	Ecological Momentary Assessment
240 minutes	7 days
▪ Assessment of psychopathology (SCID-CV, SCID-PD, and SCID-D interviews, self-report questionnaires)<br>▪ Assessment of variables used as between-level predictors (self-report questionnaires)<br>▪ Review of inclusion and exclusion criteria	▪ Micro assessment of self-report outcome variables (affective arousal and valence, and dissociation) in daily life<br>▪ Intermittent micro assessment of confounded context variables (daily functioning) in daily life

Note. This table is an adapted excerpt from Heekerens et al. (2023). SCID = Structured Clinical Interview for the Diagnostic and Statistical Manual of Mental Disorders, Fifth Edition (DSM-5): CV = Clinical Version (Beesdo-Baum et al., 2019a), PD = Personality Disorders (Beesdo-Baum et al., 2019b), D = Dissociative symptoms and disorders (Steinberg, 2022).

Table S4

Number and Percentage of Valid Responses on the Process Model of Emotion Regulation Questionnaire (Heekerens et al., 2024; N = 87)

Item number	1		2		3		4		5		6	
	N	%	N	%	N	%	N	%	N	%	N	%
Confront Unpleasant Situations	87	100	87	100	87	100	87	100	-	-	-	-
Avoid Unpleasant Situations	22	25.29	22	25.29	87	100	87	100	87	100	87	100
Resolve Conflicts	64	73.56	87	100	87	100	87	100	87	100	87	100
Avoid Conflict	87	100	87	100	87	100	87	100	87	100	-	-
Focus Elsewhere	64	73.56	64	73.56	64	73.56	64	73.56	-	-	-	-
Cognitively Distract	87	100	87	100	87	100	87	100	-	-	-	-
Consider Benefits	87	100	87	100	64	73.56	87	100	64	73.56	87	100
Reduce Importance	22	25.29	100	87	22	25.29	-	-	-	-	-	-
Emotional Sharing	100	87	100	87	100	87	-	-	-	-	-	-
Expressive Suppression	22	25.29	100	87	64	73.56	-	-	-	-	-	-

Note. PMERQ data was missing entirely for one of 88 participants. Missing responses occurred due to reformulation of the German items at the time (documented by Heekerens et al., 2024), resulting in three different version of the questionnaire.

Version one was administered with 22 individuals missing the following items: Avoid Unpleasant Situations 1 and 2, Resolve Conflicts 1, Focus Elsewhere 1, 2, 3, and 4, Consider Benefits 3 and 5, Reduce Importance 1 and 3, and Expressive Suppression 1 and 3. Version 2 was administered with 42 missing the following items: Avoid Unpleasant Situations 1 and 2, Reduce Importance 1 and 3, and Expressive Suppression 1. Version 3 was administered with 23 individuals not missing any items. All revisions involved stylistic wording only.

Table S5

Confirmatory Factor Analysis Measurement Models for the PMERQ (Heekerens et al., 2024; N=87)

Subscale	χ^2 (df)	CFI	RMSEA (90% CI)	SRMR
Confront Unpleasant Situations[0]	12.81 (2)	.873	.249 (.131, .387)	.047
Confront Unpleasant Situations[1 and 2]	0.28 (1)	1.000	.000 (.000, .229)	.007
Avoid Unpleasant Situations[0]	14.39 (9)	.940	.083 (.000, .159)	.064
Resolve Conflicts[0]	31.75 (9)	.891	.170 (.109, .237)	.046
Resolve Conflicts[5 and 6]	1.82 (8)	1.000	.000 (.000, .099)	.019
Avoid Conflict[0]	5.69 (5)	.994	.040 (.000, .158)	.031
Focus Elsewhere[0]	27.83 (2)	.364	.449 (.331, .604)	.075
Focus Elsewhere[2 and 4]	0.30 (1)	1.000	.000 (.000, .270)	.011
Cognitively Distract[0]	6.50 (5)	.987	.059 (.000, .169)	.034
Consider Benefits[0]	29.14 (9)	.868	.160 (.098, .227)	.068
Consider Benefits[2 and 5]	18.48 (8)	.931	.123 (.048, .197)	.048
Reduce Importance[0]	0.00 (0)	1.000	.000 (.000, .000)	.000
Emotional Sharing[0]	0.00 (0)	1.000	.000 (.000, .000)	.000
Expressive Suppression[0]	1.59 (1)	.922	.082 (.000, .312)	.127
Expressive Suppression[2 and 3]	0.00 (0)	1.000	.000 (.000, .000)	.000

Note. Model fit was deemed acceptable if the following criteria were met: CFI > .90; RMSEA & SRMR < .08 (Hooper & Coughlan, 2008; Hu & Bentler, 1999). RMSEA values were not used to evaluate subscale models, as the degrees of freedom are fewer than ten, which makes the index overly sensitive (Kenny et al., 2015; Shi et al., 2022). Correlated residuals were added iteratively based on modification indices until the fit criteria were satisfied. The suffices indicate the items that were correlated. Models meeting the fit criteria are shown in black; more conservative models with insufficient fit are displayed in grey. df = degrees of freedom, CFI = Comparative Fit Index, RMSEA = Root Mean Square Error of Approximation, CI= Confidence interval, SRMR = Standardised Root Mean Square Residual.

Table S6

Contemporaneous Associations Between Affective Arousal, Affective Valence, and Dissociative States in Daily Life and Habitual Use of Emotion Regulation Strategies at Baseline (N=87)

	Confront Unpleasant Situations		Avoid Unpleasant Situations		Resolve Conflicts		Avoid Conflicts		Focus Elsewhere		Cognitively Distract		Consider Benefits		Reduce Importance		Emotional Sharing		Expressive Suppression	
	Estimate	95% CI	Estimate	95% CI	Estimate	95% CI	Estimate	95% CI	Estimate	95% CI	Estimate	95% CI	Estimate	95% CI	Estimate	95% CI	Estimate	95% CI	Estimate	95% CI
Confront Unpleasant Sit.																				
Avoid Unpleasant Sit.	**-.70*****	[-.87, -.54]																		
Resolve Conflicts	.40**	[.12, .68]	-.33*	[-.60, -.05]																
Avoid Conflict	-.23	[-.48, .02]	**.46*****	[.20, .72]	-.01	[-.28, .27]														
Focus Elsewhere	.36*	[.00, .71]	-.18	[-.53, .16]	**.64*****	[.33, .94]	-.09	[-.28, .11]												
Cognitively Distract	-.28*	[-.51, -.04]	.39**	[.12, .65]	.15	[-.14, .44]	**.42*****	[.20, .65]	.12	[-.30, .53]										
Consider Benefits	**.51*****	[.30, .71]	-.21	[-.45, .03]	**.55*****	[.36, .74]	-.13	[-.36, .11]	**.79*****	[.59, .99]	.07	[-.20, .35]								
Reduce Importance	.04	[-.23, .30]	.11	[-.18, .40]	.39**	[.13, .65]	-.12	[-.39, .16]	**.60*****	[.37, .82]	.15	[-.19, .48]	.59	[.33, .86]						
Emotional Sharing	.35**	[.10, .61]	-.30**	[-.54, -.06]	**.46****	[.20, .72]	-.02	[-.32, .27]	.21	[-.30, .72]	.24	[-.05, .52]	.31	[.08, .55]	.07	[-.20, .24]				
Expressive Suppression	-.29	[-.69, .12]	.52**	[.15, .88]	-.22	[-.84, .41]	.70**	[.21, 1.00]	.06	[-.37, .49]	.44**	[.16, .72]	-.01	[-.70, .76]	.18	[-.02, .56]	-.12	[-.62, .53]		

Note. Displayed are standardised Bayesian estimates. The models use continuous predictors, linking latent scores from emotion regulation preferences at baseline to individual variability in autoregressive, contemporaneous, and cross-lagged effects between affective arousal and valence, and dissociation in daily life. 95% CI = credible interval. *p < .05, **p < .01, ***p < .001, *p* values marked in bold remained significant after applying the Holm correction.

Table S7

Comparison of Contemporaneous Interrelations between PMERQ Scales (Heekerens et al., 2024; N=87): Latent Minus Manifest Estimates

	Confront Unpleasant Sit.	Avoid Unpleasant Sit.	Resolve Conflicts	Avoid Conflicts	Focus Elsewhere	Cognitively Distract	Consider Benefits	Reduce Importance	Emotional Sharing
Avoid Unpleas. Sit.	-.11								
Resolve Conflicts	.02	-.03							
Avoid Conflicts	-.06	.07	.00						
Focus Elsewhere	.03	-.05	.14	-.14					
Cognitively Distract	-.04	.05	.07	.02	-.10				
Consider Benefits	.06	-.03	.06	-.04	.12	-.01			
Reduce Importance	.04	.06	.10	.02	.19	.04	.07		
Emotional Sharing	-.06	-.04	.01	-.03	-.14	.11	.00	.01	
Expressive Suppr.	-.10	.13	-.19	.43	.17	.07	-.03	.05	.16

Table S8

Comparison of Habitual Use of Emotion Regulation Strategies Predicting Means and Autoregressions of Affective Arousal, Affective Valence, and Dissoiciation in Daily Life for Participants with BPD (N=63) and PTSD (N=46)

	Confront Unpleasant Situations		Avoid Unpleasant Situations		Resolve Conflicts		Avoid Conflicts		Focus Elsewhere		Cognitively Distract		Consider Benefits		Reduce Importance		Emotional Sharing		Expressive Suppression	
	BPD	PTSD	BPD	PTSD	BPD	PTSD	BPD	PTSD	BPD	PTSD	BPD	PTSD	BPD	PTSD	BPD	PTSD	BPD	PTSD	BPD	PTSD
Predictive Effects on Means																				
Arousal	-0.04	-0.12	-0.04	0.03	-0.08	0.01	0.02	0.13	-0.28	-0.01	-0.12	-0.16	0.14	0.20	-0.12	0.01	-0.03	-0.14	-0.16	0.36
	[-0.35, 0.28]	[-0.43, 0.24]	[-0.37, 0.29]	[-0.34, 0.40]	[-0.39, 0.24]	[-0.36, 0.36]	[-0.29, 0.33]	[-0.25, 0.45]	[-0.61, 0.14]	[-0.47, 0.45]	[-0.43, 0.20]	[-0.48, 0.20]	[-0.18, 0.42]	[-0.18, 0.51]	[-0.52, 0.25]	[-0.41, 0.41]	[-0.34, 0.27]	[-0.47, 0.22]	[-0.62, 0.56]	[-0.63, 0.22]
Valence	-0.03	-0.07	0.18	0.00	0.06	-0.03	-0.04	-0.01	0.07	0.00	0.08	0.01	-0.01	-0.24	0.01	-0.19	0.26	0.10	-0.24	-0.27
	[-0.32, 0.28]	[-0.42, 0.30]	[-0.14, 0.47]	[-0.37, 0.39]	[-0.25, 0.35]	[-0.37, 0.33]	[-0.34, 0.26]	[-0.37, 0.34]	[-0.31, 0.44]	[-0.48, 0.48]	[-0.23, 0.38]	[-0.34, 0.35]	[-0.31, 0.28]	[-0.54, 0.12]	[-0.33, 0.34]	[-0.58, 0.30]	[-0.04, 0.53]	[-0.26, 0.43]	[-0.88, 0.39]	[-0.90, 0.60]
Derealisation	-0.20	-0.22	0.20	-0.11	-0.09	-0.10	0.17	-0.04	-0.16	-0.04	0.09	-0.27	-0.01	-0.02	-0.02	0.06	-0.18	-0.29	0.63*	0.37
	[-0.47, 0.10]	[-0.54, 0.15]	[-0.11, 0.47]	[-0.47, 0.28]	[-0.37, 0.20]	[-0.44, 0.25]	[-0.12, 0.44]	[-0.39, 0.32]	[-0.49, 0.22]	[-0.51, 0.46]	[-0.21, 0.37]	[-0.57, 0.10]	[-0.30, 0.28]	[-0.37, 0.33]	[-0.44, 0.11]	[-0.40, 0.47]	[-0.44, 0.11]	[-0.58, 0.64]	[0.15, 0.94]	[-0.53, 0.90]
Gaps in	-0.11	-0.12	0.00	0.00	-0.09	-0.13	0.01	0.01	-0.25	-0.06	-0.16	-0.24	-0.06	-0.08	0.06	0.01	-0.11	-0.40*	0.35	0.29
Awareness	[-0.39, 0.20]	[-0.47, 0.26]	[-0.31, 0.30]	[-0.38, 0.39]	[-0.38, 0.21]	[-0.46, 0.24]	[-0.29, 0.30]	[-0.35, 0.36]	[-0.56, 0.14]	[-0.53, 0.49]	[-0.44, 0.14]	[-0.55, 0.14]	[-0.35, 0.24]	[0.43, 0.29]	[-0.43, 0.49]	[-0.42, 0.44]	[-0.39, 0.19]	[-0.67, -0.06]	[-0.19, 0.88]	[-0.48, 0.89]
Predictive Effects on Autoregressions																				
Arousal	-0.13	-0.04	0.14	-0.05	0.12	0.28	-0.20	-0.28	0.03	0.04	-0.02	0.09	-0.06	0.27	0.24	0.45	-0.20	0.04	0.24	-0.31
	[-0.45, 0.21]	[-0.48, 0.38]	[-0.22, 0.46]	[-0.48, 0.40]	[-0.21, 0.43]	[-0.19, 0.64]	[-0.50, 0.14]	[-0.67, 0.17]	[-0.36, 0.40]	[-0.65, 0.67]	[-0.35, 0.32]	[-0.36, 0.51]	[-0.37, 0.28]	[-0.18, 0.63]	[-0.13, 0.57]	[-0.07, 0.83]	[-0.51, 0.14]	[-0.39, 0.44]	[-0.34, 0.70]	[-0.88, 0.50]
Valence	-0.06	-0.05	0.30	-0.00	0.29	0.32	0.08	0.00	0.07	0.01	-0.00	0.13	0.12	0.17	0.18	0.40	-0.08	0.01	0.05	-0.14
	[-0.37, 0.27]	[-0.45, 0.35]	[-0.03, 0.58]	[-0.39, 0.40]	[-0.04, 0.56]	[-0.08, 0.64]	[-0.23, 0.38]	[-0.39, 0.38]	[-0.29, 0.41]	[-0.49, 0.50]	[-0.32, 0.32]	[-0.30, 0.51]	[-0.20, 0.42]	[-0.23, 0.52]	[-0.56, 0.60]	[-0.40, 0.75]	[-0.39 0.25]	[-0.38, 0.38]	[-0.50, 0.59]	[-0.79, 0.72]
Derealisation	-0.20	-0.16	0.06	0.00	0.04	0.12	-0.24	-0.23	-0.11	0.02	-0.07	-0.11	-0.02	-0.04	-0.13	0.20	-0.09	0.09	0.32	-0.09
	[-0.48, 0.12]	[-0.52, 0.24]	[-0.27, 0.38]	[-0.40 0.40]	[-0.28, 0.35]	[-0.28, 0.48]	[-0.51, 0.07]	[-0.56, 0.15]	[-0.48, 0.28]	[-0.43, 0.46]	[-0.37, 0.25]	[-0.47, 0.28]	[-0.32, 0.29]	[-0.40, 0.34]	[-0.52, 0.43]	[-0.59, 0.64]	[-0.39, 0.23]	[-0.29, 0.45]	[-0.18, 0.75]	[-0.75, 0.58]
Gaps in	-0.10	-0.02	0.13	0.06	-0.20	0.00	-0.28	-0.08	-0.15	-0.00	-0.35*	-0.13	-0.20	-0.12	-0.01	-0.06	-0.32*	-0.15	0.25	-0.05
Awareness	[-0.48, 0.13]	[-0.41, 0.37]	[-0.20, 0.44]	[-0.34, 0.45]	[-0.48, 0.12]	[-0.37, 0.38]	[-0.54, 0.02]	[-0.43, 0.30]	[-0.51, 0.25]	[-0.45, 0.45]	[-0.60, -0.05]	[-0.50, 0.28]	[-0.48, 0.24]	[-0.48, 0.27]	[-0.39, 0.36]	[-0.54, 0.46]	[-0.59, -0.01]	[-0.52, 0.26]	[-0.33, 0.80]	[-0.74, 0.69]

Note. 12 Participants were diagnosed with both BPD and PTSD. We did not conduct analyses for the four participants with a DD but not BPD or PTSD diagnosis. Displayed are standardised Bayesian estimates and 95% credibility intervals. The models use continuous predictors, linking latent scores from habitual use of emotion regulation strategies at baseline to means and individual variability in autoregressive effects of affective arousal and valence, and dissociation in daily life. BPD = borderline personality disorder; DD = dissociative disorders; PTSD = posttraumatic stress disorder; CI = credibility interval. *p < .05 (uncorrected *p* value).

Table S9

Multilevel Reliability Coefficients Alpha for Affective Arousal, Affective Valence, and Dissociation in Daily Life on Within- and Between-person Level, and for the Overall Composite Score (N=88)

	α_{2level}		α_{within}		$\alpha_{between}$	
	Estimate	95% CI	Estimate	95% CI	Estimate	95% CI
Arousal	.86	[.84, .87]	.83	[.82, .83]	.90	[.87, .93]
Valence	.87	[.85, .88]	.82	[.82, .83]	.93	[.91, .95]
Derealisation	.88	[.87, .89]	.83	[.82, .84]	.94	[.92, .96]
Gaps in Awareness	.84	[.82, .86]	.80	[.79, .81]	.89	[.85, .93]

Note. α_{2level} = overall composite score reliability, α_{within} = within-person reliability, $\alpha_{between}$ = between-person reliability.

Table S10

Results of the Temporal Associations Between Affective Arousal, Affective Valence, and Dissociation from Heekerens, Gross et al. (2026)

	Derealisation (N=87)		Gaps in Awareness (N=86)		Arousal (N=87)		Valence (N=87)	
	Estimate	95% CI	Estimate	95% CI	Estimate	95% CI	Estimate	95% CI
				Unstandardised Parameter Estimates				
Fixed Effects (Means)								
$\gamma_{\varphi\text{\_Affect\_t-1}\rightarrow\text{Affect\_t}}$	0.73*	[0.68, 0.77]	0.70*	[0.66, 0.75]	0.82*	(0.78, 0.86]	0.80*	[0.76, 0.84]
$\gamma_{\varphi\text{\_Dissociation\_t-1}\rightarrow\text{ Dissociation \_t}}$	0.64*	[0.59, 0.70]	0.65*	[0.59, 0.70]	0.65*	[0.59, 0.70]	0.64*	[0.58, 0.70]
$\gamma_{\varphi\text{\_Affect\_t-1}\rightarrow\text{ Dissociation \_t}}$	0.05*	[0.03, 0.07]	0.03*	[0.01, 0.06]	-0.04*	1-0.06, -0.011	-0.04*	[-0.06, -0.01]
$\gamma_{\varphi\text{\_Dissociation\_t-1}\rightarrow\text{ Affect \_t}}$	0.05	(-0.03, 0.13]	0.11*	[0.02, 0.19]	0.03	-O.03, 0.09]	-0.04	[-0.10, 0.03]
Random Effects (Variances)								
$\varphi_{\text{Affect\_t-1}\rightarrow\text{Affect\_t}}$	0.03*	[0.02, 0.05]	0.03*	[0.02, 0.04]	0.02*	[0.01, 0.03]	0.02*	10.01, 0.03]
$\varphi_{\text{Dissociation\_t-1}\rightarrow\text{ Dissociation \_t}}$	0.04*	[0.02, 0.06]	0.04*	[0.03, 0.071	0.04*	[0.02, 0.06]	0.04*	[0.03, 0.07]
$\varphi_{\text{Affect\_t-1}\rightarrow\text{ Dissociation \_t}}$	**0.00**	**[0.00,0.01]**	**0.00**	**[0.00, 0.011**	**0.00**	**10.00, 0.01]**	**0.00**	**[0.00, 0.01]**
$\varphi_{\text{Dissociation\_t-1}\rightarrow\text{ Affect \_t}}$	**0.04***	**[0.01,0.08]**	**0.05***	**[0.02, 0.10]**	**0.02***	**10.01, 0.04]**	**0.02***	**[0.01, 0.05]**
				Standardised Parameter Estimates				
Within-Person Standardisation (Averaged Across Clusters)								
$\varphi_{\text{Affect\_t-1}\rightarrow\text{Affect\_t}}$	.73*	[0.70, 0.76]	.70*	[0.68, 0.73]	.82*	[0.80, 0.84]	.80*	[0.78, 0.83]
$\varphi_{\text{Dissociation\_t-1}\rightarrow\text{ Dissociation \_t}}$	.64*	[0.61, 0.67]	.64*	[0.60, 0.68]	.65%	[0.62, 0.69]	.64*	10.60, 0.68]
$\varphi_{\text{Affect\_t-1}\rightarrow\text{ Dissociation \_t}}$	**.09***	**[0.07, 0.13]**	**.07%**	**[0.04, 0.11]**	**-.07***	**[-0.10, -0.03]**	**-.08***	**[-0.10, -0.05]**
$\varphi_{\text{Dissociation\_t-1}\rightarrow\text{ Affect \_t}}$	**.03**	**[0.00, 0.06]**	**.06***	**[0.03, 0.09]**	**.01**	**[-0.02, 0.04]**	**-.03**	**[-0.05, 0.00]**

Note. This table has been derived from Heekerens, Gross et al. (2026), with kind permission to be reused for this study. All parameters denote posterior medians. We bold parameters relevant to our hypothesis tests. The 95% CIs denote Bayesian credibility intervals. Affective states of arousal and valence were assessed on 0-6 scales, while dissociative states of derealization and gaps in awareness were assessed on 0-4 scales. All models include latent variables measured with two indicators each. The interval between two measurements was approximately 15 minutes. μ_{Affect} = person-specific mean of affective state across measurement occasions, $\mu_{\text{Dissociation}}$ = person-specific mean of dissociative state across measurement occasions, $\gamma_{\phi\text{Affect}}$ = average autoregressive effect (fixed effect) of affective state, $\gamma_{\phi\text{Dissociation}}$ = average autoregressive effect (fixed effect) of dissociative state, $\gamma_{\phi\text{Affect\_t−1}\rightarrow\text{Dissociation\_t}}$ = average slope (fixed effect) of the regressions of dissociative state at time t on affective state at time t-1, $\gamma_{\phi\text{Dissociation\_t−1}\rightarrow\text{Affect\_t}}$ = average slope (fixed effect) of the regressions of affective state at time t on dissociative state at time t-1, ϕ_{Affect} = random, person-specific autoregressive effect of affective state, $\phi_{\text{Dissociation}}$ = random, person-specific autoregressive effect of dissociative state, $\phi_{\text{Affect\_t−1}\rightarrow\text{Dissociation\_t}}$ = random, person-specific slope of the regressions of dissociative state at time t on affective state at time t-1, $\phi_{\text{Dissociation\_t−1}\rightarrow\text{Affect\_t}}$ = random, person-specific slope of the regressions of affective state at time t on dissociative state at time t-1. $^*p < .05$

Table S11

Holm Correction for Test Family Prediction of Means (N =40 Tests)

Prediction of Means																				
Predictor	Arousal					Valence					Derealisation					Gaps in Awareness				
	Estimate	*p*	Rank	Multiplier	*p* (adj.)	Estimate	*p*	Rank	Multiplier	*p* (adj.)	Estimate	*p*	Rank	Multiplier	*p* (adj.)	Estimate	*p*	Rank	Multiplier	*p* (adj.)
Confront Unpleasant Situations	0.01	0.968	37	4	1.000	-0.06	0.664	22	19	1.000	-0.08	0.518	14	27	1.000	-0.07	0.614	19	22	1.000
Avoid Unpleasant Situations	-0.02	0.892	32	9	1.000	0.14	0.292	9	32	1.000	0.08	0.534	15	26	1.000	0.05	0.710	24	17	1.000
Resolve Conflicts	-0.15	0.252	8	33	1.000	0.10	0.438	13	28	1.000	-0.16	0.196	6	35	1.000	-0.18	0.154	3	38	1.000
Avoid Conflicts	0.02	0.894	33	8	1.000	-0.00	0.99	39	2	1.000	0.06	0.620	20	21	1.000	-0.04	0.786	30	11	1.000
Focus Elsewhere	-0.02	0.926	34	7	1.000	-0.09	0.598	18	23	1.000	-0.05	0.768	27	14	1.000	-0.10	0.540	16	25	1.000
Cognitively Distract	-0.03	0.806	31	10	1.000	-0.01	0.944	36	5	1.000	-0.05	0.670	23	18	1.000	-0.16	0.216	7	34	1.000
Consider Benefits	0.18	0.168	4	37	1.000	-0.10	0.436	12	29	1.000	0.04	0.750	26	15	1.000	-0.04	0.770	28	13	1.000
Reduce Importance	-0.01	0.978	38	3	1.000	-0.00	0.996	40	1	1.000	-0.02	0.934	35	6	1.000	0.06	0.780	29	12	1.000
Emotional Sharing	-0.06	0.648	21	20	1.000	0.18	0.172	5	36	1.000	-0.25†	0.042	1	40	1.000	-0.19	0.124	2	39	1.000
Expressive Suppression	-0.08	0.748	25	16	1.000	-0.14	0.576	17	24	1.000	0.22	0.358	11	30	1.000	0.24	0.308	10	31	1.000

Note. No associations were statistically significant after applying the Holm correction for multiple comparisons. Mean values were determined as individual averages over all EMA measurements. Displayed are standardised Bayesian estimates. *p* (adj.) = Holm-adjusted *p* value.

Table S12

Holm Correction for Test Family Prediction of Autoregressions (N =40 Tests)

Prediction of Autoregressions																				
Predictor	Arousal$_{(t-1)}$ → Arousal$_{(t)}$					Valence$_{(t-1)}$ → Valence$_{(t)}$					Derealisation$_{(t-1)}$ → Derealisation$_{(t)}$					Gaps$_{(t-1)}$ → Gaps$_{(t)}$				
	Estimate	*p*	Rank	Multiplier	*p* (adj.)	Estimate	*p*	Rank	Multiplier	*p* (adj.)	Estimate	*p*	Rank	Multiplier	*p* (adj.)	Estimate	*p*	Rank	Multiplier	*p* (adj.)
Confront Unpleasant Situations	-0.15	0.298	12	29	1.000	-0.12	0.396	16	25	1.000	-0.18	0.184	7	34	1.000	-0.07	0.250	10	31	1.000
Avoid Unpleasant Situations	0.05	0.764	32	9	1.000	0.17	0.248	9	32	1.000	0.06	0.686	29	12	1.000	0.05	0.678	28	13	1.000
Resolve Conflicts	0.20	0.176	6	35	1.000	0.30†	0.034	1	40	1.000	0.03	0.832	35	6	1.000	-0.18	0.530	21	20	1.000
Avoid Conflicts	-0.13	0.378	14	27	1.000	0.07	0.610	24	17	1.000	-0.15	0.286	11	30	1.000	-0.04	0.216	8	33	1.000
Focus Elsewhere	0.09	0.636	26	15	1.000	0.10	0.536	22	19	1.000	-0.13	0.454	18	23	1.000	-0.10	0.740	30	11	1.000
Cognitively Distract	0.0	0.986	39	2	1.000	0.00	0.996	40	1	1.000	-0.06	0.660	27	14	1.000	-0.16	0.062	4	37	1.000
Consider Benefits	-0.02	0.892	38	3	1.000	0.09	0.528	20	21	1.000	-0.11	0.440	17	24	1.000	-0.04	0.122	5	36	1.000
Reduce Importance	-0.22	0.472	19	22	1.000	0.15	0.794	33	8	1.000	-0.05	0.878	36	5	1.000	0.06	0.888	37	4	1.000
Emotional Sharing	-0.28	0.052	2	39	1.000	-0.12	0.388	15	26	1.000	-0.09	0.536	23	18	1.000	-0.19	0.058	3	38	1.000
Expressive Suppression	0.25	0.326	13	28	1.000	0.06	0.798	34	7	1.000	0.12	0.622	25	16	1.000	0.24	0.760	31	10	1.000

Note. No associations were statistically significant after applying the Holm correction for multiple comparisons. Displayed are standardised Bayesian estimates. *p* (adj.) = Holm-adjusted *p* value.

Table S13

Holm Correction for Test Family Prediction of Contemporaneous Cross-Regressions (N =40 Tests)

	**Prediction of Contemporaneous Cross-Regressions**																			
Predictor	$Arousal_{(t)} \leftrightarrow Derealisation_{(t)}$					$Arousal_{(t)} \leftrightarrow Gaps_{(t)}$					$Valence_{(t)} \leftrightarrow Derealisation_{(t)}$					$Valence_{(t)} \leftrightarrow Gaps_{(t)}$				
	Estimate	*p*	Rank	Multiplier	*p* (adj.)	Estimate	*p*	Rank	Multiplier	*p* (adj.)	Estimate	*p*	Rank	Multiplier	*p* (adj.)	Estimate	*p*	Rank	Multiplier	*p* (adj.)
Confront Unpleasant Situations	0.11	0.432	9	32	1.000	0.12	0.428	8	33	1.000	0.00	0.998	40	1	1.000	-0.04	0.770	23	18	1.000
Avoid Unpleasant Situations	-0.16	0.290	4	37	1.000	-0.25	0.102	2	39	1.000	-0.02	0.922	30	11	1.000	0.05	0.750	22	19	1.000
Resolve Conflicts	0.01	0.926	32	9	1.000	0.01	0.972	39	2	1.000	0.02	0.892	28	13	1.000	-0.05	0.720	20	21	1.000
Avoid Conflicts	-0.13	0.370	6	35	1.000	-0.27	0.051	28	13	1.000	0.05	0.744	20	21	1.000	0.12	0.402	6	35	1.000
Focus Elsewhere	-0.12	0.472	10	31	1.000	0.02	0.902	29	12	1.000	0.16	0.338	5	36	1.000	-0.03	0.876	27	14	1.000
Cognitively Distract	0.01	0.930	34	7	1.000	-0.07	0.626	15	26	1.000	0.05	0.716	19	22	1.000	0.08	0.580	12	29	1.000
Consider Benefits	0.01	0.924	31	10	1.000	-0.03	0.812	24	17	1.000	0.06	0.662	17	24	1.000	0.07	0.624	14	27	1.000
Reduce Importance	0.04	0.944	36	5	1.000	-0.07	0.940	35	6	1.000	0.04	0.958	38	3	1.000	0.02	0.928	33	8	1.000
Emotional Sharing	0.16	0.286	3	38	1.000	0.09	0.530	11	30	1.000	0.01	0.952	37	4	1.000	-0.06	0.680	18	23	1.000
Expressive Suppression	0.06	0.834	25	16	1.000	0.15	0.644	16	25	1.000	0.06	0.848	26	15	1.000	0.17	0.594	13	28	1.000

Note. No associations were statistically significant after applying the Holm correction for multiple comparisons. Displayed are standardised Bayesian estimates. *p* (adj.) = Holm-adjusted *p* value.

Table S14

Holm Correction for Test Family Cross-Lagged Regressions (N=80 Tests)

Prediction of Cross-Lagged Regressions

Predictor	Arousal$_{(t-1)}$ → Derealisation$_{(t)}$					Derealisation$_{(t-1)}$ → Arousal$_{(t)}$					Arousal$_{(t-1)}$ → Gaps$_{(t)}$					Gaps$_{(t-1)}$ → Arousal$_{(t)}$				
	Estimate	*p*	Rank	Multiplier	*p* (adj.)	Estimate	*p*	Rank	Multiplier	*p* (adj.)	Estimate	*p*	Rank	Multiplier	*p* (adj.)	Estimate	*p*	Rank	Multiplier	*p* (adj.)
Confront Unpleasant Sit.	-0.04	0.850	54	27	1.000	-0.02	0.892	60	21	1.000	0.04	0.600	24	57	1.000	0.08	0.652	28	53	1.000
Avoid Unpleasant Sit.	0.00	0.994	77	4	1.000	-0.04	0.844	53	28	1.000	-0.16	0.812	47	34	1.000	-0.05	0.430	13	68	1.000
Resolve Conflicts	-0.11	0.584	22	59	1.000	0.06	0.766	39	42	1.000	-0.21	0.262	4	77	1.000	0.04	0.860	55	26	1.000
Avoid Conflicts	-0.10	0.606	25	56	1.000	-0.04	0.802	43	38	1.000	-0.09	0.678	30	51	1.000	0.18	0.388	12	69	1.000
Focus Elsewhere	0.03	0.912	62	19	1.000	0.00	0.996	78	3	1.000	0.30	0.134	2	79	1.000	-0.22	0.206	3	78	1.000
Cognitively Distract	0.19	0.366	10	71	1.000	-0.15	0.358	9	72	1.000	-0.06	0.734	35	46	1.000	-0.06	0.718	34	47	1.000
Consider Benefits	-0.05	0.810	46	35	1.000	-0.04	0.828	49	32	1.000	0.00	0.986	74	7	1.000	-0.06	0.742	36	45	1.000
Reduce Importance	0.11	0.768	40	41	1.000	0.07	0.804	44	37	1.000	0.01	0.962	70	11	1.000	0.26	0.532	17	64	1.000
Emotional Sharing	0.31	0.106	1	80	1.000	-0.04	0.834	52	29	1.000	0.06	0.828	50	31	1.000	0.14	0.574	20	61	1.000
Expressive Suppression	0.07	0.982	73	8	1.000	-0.01	0.816	48	33	1.000	0.06	0.828	51	30	1.000	0.14	0.574	21	60	1.000

Predictor	Valence$_{(t-1)}$ → Derealisation$_{(t)}$					Derealisation$_{(t-1)}$ → Valence$_{(t)}$					Valence$_{(t-1)}$ → Gaps$_{(t)}$					Gaps$_{(t-1)}$ → Valence$_{(t)}$				
	Estimate	*p*	Rank	Multiplier	*p* (adj.)	Estimate	*p*	Rank	Multiplier	*p* (adj.)	Estimate	*p*	Rank	Multiplier	*p* (adj.)	Estimate	*p*	Rank	Multiplier	*p* (adj.)
Confront Unpleasant Sit.	-0.08	0.648	27	54	1.000	0.00	0.992	75	6	1.000	0.03	0.888	58	23	1.000	0.00	0.996	79	2	1.000
Avoid Unpleasant Sit.	0.01	0.962	71	10	1.000	0.05	0.780	41	40	1.000	0.19	0.366	11	70	1.000	-0.12	0.548	18	63	1.000
Resolve Conflicts	0.05	0.806	45	36	1.000	-0.02	0.916	63	18	1.000	0.11	0.560	19	62	1.000	0.10	0.586	23	58	1.000
Avoid Conflicts	0.00	0.996	80	1	1.000	0.03	0.888	59	22	1.000	0.23	0.862	56	25	1.000	-0.03	0.862	57	24	1.000
Focus Elsewhere	0.07	0.710	33	48	1.000	0.14	0.510	16	65	1.000	0.11	0.490	14	67	1.000	0.15	0.490	15	66	1.000
Cognitively Distract	0.08	0.690	32	49	1.000	0.02	0.904	61	20	1.000	-0.07	0.352	7	74	1.000	0.17	0.352	8	73	1.000
Consider Benefits	0.00	0.992	76	5	1.000	-0.01	0.980	72	9	1.000	0.02	0.934	66	15	1.000	-0.02	0.934	67	14	1.000
Reduce Importance	0.28	0.262	5	76	1.000	0.10	0.682	31	50	1.000	0.01	0.938	68	13	1.000	0.02	0.938	69	12	1.000
Emotional Sharing	-0.09	0.610	26	55	1.000	-0.05	0.798	42	39	1.000	0.02	0.926	65	16	1.000	0.19	0.284	6	75	1.000
Expressive Suppression	-0.09	0.748	37	44	1.000	0.03	0.924	64	17	1.000	0.13	0.652	29	52	1.000	-0.09	0.750	38	43	1.000

Note. No associations were statistically significant after applying the Holm correction for multiple comparisons. Displayed are standardised Bayesian estimates. CI = credibility interval; *p* (adj.) = Holm-adjusted *p* value.

Summary of Model Convergence Diagnostics

Convergence diagnostics were evaluated across three model classes: prediction of means and autoregressions (Table S15), prediction of contemporaneous associations (Table S16), and prediction of cross-lagged associations (Table S17). Convergence was assessed using the potential scale reduction (PSR) factor, effective sample size (ESS), and the number of clusters exhibiting unstable estimation during sampling. All model estimations terminated normally.

Models predicting means/autoregressions and contemporaneous associations converged well overall, with final PSR values close to 1.00 (predominantly ≤ 1.02), ESS numbers generally exceeding several hundred draws, and few to no clusters showing estimation instability. These results indicate stable and reliable posterior estimation for both model classes.

In contrast, models predicting cross-lagged associations, while still displaying acceptable convergence according to the main criterion (PSR < 1.10), converged worse than the means/autoregression and contemporaneous association models. This was reflected in elevated final PSR values relative to the other model classes, lower ESS values (often below 200), and a higher number of clusters showing unstable estimation (up to 20 in some models). This pattern likely reflects the greater estimation complexity inherent to lagged parameters in intensive longitudinal designs, and suggests that findings from the cross-lagged models should be interpreted more cautiously compared to the other classes of models.

Across all three model classes, models including *Reduced Importance* as a predictor also displayed acceptable convergence according to PSR (< 1.10), but converged comparatively worse than models with other predictors, as indicated by relatively elevated PSR values, markedly lower effective sample sizes, and increased cluster instability. This suggests that estimates involving the reduced importance predictor should be interpreted with somewhat greater caution throughout the reported findings.

Table S15

Summary of Converge Parameters for Models Predicting Means and Autoregressions

Prediction of Means and Autoregressions																
Predictor	Arousal/Arousal$_{(t-1)}$ → Arousal$_{(t)}$				Valence/Valence$_{(t-1)}$ → Valence$_{(t)}$				Derealisation/Derealisation$_{(t-1)}$ → Derealisation$_{(t)}$				Gaps/Gaps$_{(t-1)}$ → Gaps$_{(t)}$			
	Final PSR	Max PSR	Min ESS	N Unst. Clu.	Final PSR	Max PSR	Min ESS	N Unst. Clu.	Final PSR	Max PSR	Min ESS	N Unst. Clu.	Final PSR	Max PSR	Min ESS	N Unst. Clu.
Confront Unpleasant Situations	1	1.117	1300	0	1.003	1.792	914	2	1.001	1.19	1513	0	1.001	1.423	992	0
Avoid Unpleasant Situations	1.001	1.315	1212	0	1.003	1.173	670	1	1.002	1.212	1323	0	1.001	1.207	1128	0
Resolve Conflicts	1	1.731	1398	0	1.001	1.486	829	1	1.001	1.305	1378	0	1.002	1.256	1076	0
Avoid Conflicts	1.002	1.419	1240	1	1	1.267	892	1	1.001	1.12	1417	0	1	1.186	1171	0
Focus Elsewhere	1.001	1.186	1225	0	1.002	1.392	910	0	1.002	1.409	1275	0	1.002	1.275	1065	0
Cognitively Distract	1.003	1.505	1240	1	1.003	1.411	910	1	1.001	1.156	1443	0	1.001	1.074	977	0
Consider Benefits	1.002	1.301	1252	1	1.001	1.356	883	1	1.002	1.272	1335	0	1	1.193	1229	0
Reduce Importance	1.002	1.748	21	0	1.043	2.536	1415	1	1.041	1.916	21	0	1.001	1.537	27	0
Emotional Sharing	1.002	1.236	1171	1	1.001	1.4	783	1	1	1.107	1429	1	1.001	1.176	1034	0
Expressive Suppression	1	1.244	1195	1	1.002	1.29	853	0	1.001	1.448	1320	0	1.003	1.115	1130	0

Note. All estimations terminated normally. Final PSR = Potential Scale Reduction Factor at the end of model estimation; Max PSR = highest Potential Scale Reduction Factor during the run; Min ESS = lowest Effective Sample Size of all parameters; N Unst. Ch. = Number of Unstable Clusters.

Table S16

Summary of Converge Parameters for Models Predicting Contemporaneous Cross-Regressions

Prediction of Contemporaneous Cross-Regressions																
Predictor	Arousal$_{(t)}$ ↔ Derealisation$_{(t)}$				Arousal$_{(t)}$ ↔ Gaps$_{(t)}$				Valence$_{(t)}$ ↔ Derealisation$_{(t)}$				Valence$_{(t)}$ ↔ Gaps$_{(t)}$			
	Final PSR	Max PSR	Min ESS	N Unst. Clu.	Final PSR	Max PSR	Min ESS	N Unst. Clu.	Final PSR	Max PSR	Min ESS	N Unst. Clu.	Final PSR	Max PSR	Min ESS	N Unst. Clu.
Confront Unpleasant Situations	1.004	1.845	497	0	1.005	1.44	426	0	1.004	1.417	356	2	1.006	1.432	406	3
Avoid Unpleasant Situations	1.002	1.689	329	0	1.004	1.671	587	0	1.003	1.456	440	2	1.003	1.686	387	1
Resolve Conflicts	1.003	1.465	412	0	1.008	2.168	400	0	1.006	1.535	324	0	1.011	1.418	337	1
Avoid Conflicts	1.003	1.387	423	0	1.005	1.776	452	0	1.007	1.392	381	2	1.003	1.422	283	1
Focus Elsewhere	1.004	1.539	376	0	1.011	1.608	444	0	1.003	1.488	351	1	1.005	1.542	148	2
Cognitively Distract	1.004	1.543	464	0	1.008	1.697	461	0	1.004	1.378	381	1	1.004	1.605	290	2
Consider Benefits	1.003	1.44	481	0	1.003	1.538	396	0	1.002	1.564	380	1	1.004	1.49	386	2
Reduce Importance	1.012	5.069	22	1	1.044	4.81	23	0	1.044	5.234	21	1	1.041	5.041	63	1
Emotional Sharing	1.004	1.634	422	0	1.016	1.742	356	0	1.003	1.361	436	2	1.006	1.616	363	2
Expressive Suppression	1.003	1.548	492	0	1.005	2.456	537	0	1.003	1.422	549	2	1.007	1.627	338	2

Note. All estimations terminated normally. Final PSR = Potential Scale Reduction Factor at the end of model estimation; Max PSR = highest Potential Scale Reduction Factor during the run; Min ESS = lowest Effective Sample Size of all parameters; N Unst. Ch. = Number of Unstable Clusters.

Table S17

Summary of Converge Parameters for Models Predicting Cross-Lagged Regressions

Predictor	Prediction of Cross-Lagged Regressions															
	Arousal ⟷ Derealisation				Arousal ⟷ Gaps				Valence ⟷ Derealisation				$Valence_{(t)}$ ⟷ $Gaps_{(t)}$			
	Final PSR	Max PSR	Min ESS	N Unst. Clu.	Final PSR	Max PSR	Min ESS	N Unst. Clu.	Final PSR	Max PSR	Min ESS	N Unst. Clu.	Final PSR	Max PSR	Min ESS	N Unst. Clu.
Confront Unpleasant Sit.	1.015	2.016	70	4	1.022	1.759	67	7	1.046	1.591	76	14	1.028	1.697	69	13
Avoid Unpleasant Sit.	1.044	1.667	144	6	1.012	2.045	83	11	1.049	1.671	66	15	1.041	1.533	102	17
Resolve Conflicts	1.049	1.841	144	4	1.022	2.54	74	10	1.045	1.805	108	14	1.015	2.123	110	16
Avoid Conflicts	1.01	1.818	114	6	1.03	2.009	83	7	1.048	2.235	95	15	1.038	1.78	57	19
Focus Elsewhere	1.018	2.073	68	4	1.023	1.858	102	6	1.026	1.706	163	17	1.049	1.927	335	17
Cognitively Distract	1.021	2.146	89	5	1.034	1.929	56	8	1.048	2.01	83	13	1.043	2.091	70	18
Consider Benefits	1.046	1.842	137	5	1.049	2.257	132	10	1.041	2.137	97	18	1.028	2.517	84	20
Reduce Importance	1.043	5.064	21	3	1.049	2.226	22	9	1.037	1.78	45	10	1.049	3.117	21	19
Emotional Sharing	1.023	1.911	134	5	1.017	1.797	101	10	1.047	2.139	73	13	1.023	2.042	91	16
Expressive Suppression	1.019	1.58	98	4	1.016	1.993	116	6	1.048	1.683	109	16	1.024	1.62	74	18

Note. All estimations terminated normally. Final PSR = Potential Scale Reduction Factor at the end of model estimation; Max PSR = highest Potential Scale Reduction Factor during the run; Min ESS = lowest Effective Sample Size of all parameters; N Unst. Ch. = Number of Unstable Clusters.

References

Beesdo-Baum, K., Zaudig, M., & Wittchen, H.-U. (2019a). *SCID-5-CV: strukturiertes klinisches Interview für DSM-5-Störungen-Klinische Version: deutsche Bearbeitung des Structured Clinical Interview for DSM-5 Disorders-Clinician version von Michael B. First, Janet BW Williams, Rhonda S. Karg, Robert L. Spitzer*. Hogrefe.

Beesdo-Baum, K., Zaudig, M., & Wittchen, H.-U. (2019b). *SCID-5-PD: strukturiertes Klinisches Interview für DSM-5-Persönlichkeitsstörungen: deutsche Bearbeitung des Structured Clinical Interview for DSM-5-Personality Disorders von Michael B. First, Janet BW Williams, Lorna Smith Benjamin, Robert L. Spitzer*. Hogrefe.

Gross, J. J. (2015). Emotion Regulation: Current Status and Future Prospects. *Psychological Inquiry*, *26*(1), 1-26. https://doi.org/10.1080/1047840X.2014.940781

Heekerens, J. B., Gross, J. J., Kreibig, S. D., Ober, H. L., Eichentopf, R., Dittrich, T., Lebovitz, J. G., Hellmann-Regen, J., Wingenfeld, K., & Roepke, S. (2026). Does dissociation have an emotion regulation function? Evidence from daily life and the laboratory. *Clinical Psychological Science*. https://doi.org/10.1177/21677026251396803

Heekerens, J. B., Gross, J. J., Kreibig, S. D., Wingenfeld, K., & Roepke, S. (2023). The temporal dynamics of dissociation: protocol for an ecological momentary assessment and laboratory study in a transdiagnostic sample. *BMC psychology*, *11*(1), 178. https://doi.org/10.1186/s40359-023-01209-z

Heekerens, J. B., Olderbak, S., Gross, J. J., Wingenfeld, K., & Roepke, S. (2024). The Process Model of Emotion Regulation Scale (PMERQ): Psychometric properties and validity of a German version. *European Journal of Psychological Assessment*. https://doi.org/10.1027/1015-5759/a000863

Hooper, D., & Coughlan, J. (2008). Structural Equation Modelling: Guidelines for Determining Model Fit. *Electronic Journal of Business Research Methods*, *6*(1), 53-60. https://doi.org/10.21427/D7CF7R

Hu, L. t., & Bentler, P. M. (1999). Cutoff criteria for fit indexes in covariance structure analysis: Conventional criteria versus new alternatives. *Structural Equation Modeling: A Multidisciplinary Journal*, *6*(1), 1-55. https://doi.org/10.1080/10705519909540118

Kenny, D. A., Kaniskan, B., & McCoach, D. B. (2015). The Performance of RMSEA in Models With Small Degrees of Freedom. *Sociological Methods & Research*, *44*(3), 486-507. https://doi.org/10.1177/0049124114543236

Olderbak, S., Uusberg, A., MacCann, C., Pollak, K. M., & Gross, J. J. (2022). The Process Model of Emotion Regulation Questionnaire: Assessing Individual Differences in Strategy Stage and Orientation. *Assessment*, *30*(7), 2090-2114. https://doi.org/10.1177/10731911221134601

Shi, D., DiStefano, C., Maydeu-Olivares, A., & Lee, T. (2022). Evaluating SEM Model Fit with Small Degrees of Freedom. *Multivariate Behavioral Research*, *57*(2-3), 179-207. https://doi.org/10.1080/00273171.2020.1868965

Steinberg, M. (2022). *The SCID-D interview: Dissociation assessment in therapy, forensics, and research*. American Psychiatric Publications.
